# Revised Species Delimitation in the Giant Water Lily Genus *Victoria* (Nymphaeaceae) Confirms a New Species and Has Implications for Its Conservation

**DOI:** 10.3389/fpls.2022.883151

**Published:** 2022-07-04

**Authors:** Lucy T. Smith, Carlos Magdalena, Natalia A. S. Przelomska, Oscar A. Pérez-Escobar, Darío G. Melgar-Gómez, Stephan Beck, Raquel Negrão, Sahr Mian, Ilia J. Leitch, Steven Dodsworth, Olivier Maurin, Gaston Ribero-Guardia, César D. Salazar, Gloria Gutierrez-Sibauty, Alexandre Antonelli, Alexandre K. Monro

**Affiliations:** ^1^Royal Botanic Gardens, Kew, Richmond, United Kingdom; ^2^National Museum of Natural History, Smithsonian Institution, Washington, DC, United States; ^3^Herbario German Coimbra Sanz, Jardín Botánico Municipal de Santa Cruz de la Sierra, Santa Cruz de la Sierra, Bolivia; ^4^Herbario Nacional de Bolivia, Universidad Mayor de San Andrés, La Paz, Bolivia; ^5^School of Biological Sciences, University of Portsmouth, Portsmouth, United Kingdom; ^6^La Rinconada Ecoparque, Santa Cruz, Urbari, Bolivia; ^7^Calle 11 Norte #24, Urbari, Bolivia; ^8^Gothenburg Global Biodiversity Centre, Department of Biological and Environmental Sciences, University of Gothenburg, Gothenburg, Sweden; ^9^Department of Plant Sciences, University of Oxford, Oxford, United Kingdom

**Keywords:** *Victoria*, heuristic species concept, morphology, population genomics, Victorian era, Mamoré River, molecular diagnosis of species, divergence times

## Abstract

Reliably documenting plant diversity is necessary to protect and sustainably benefit from it. At the heart of this documentation lie species concepts and the practical methods used to delimit taxa. Here, we apply a total-evidence, iterative methodology to delimit and document species in the South American genus *Victoria* (Nymphaeaceae). The systematics of *Victoria* has thus far been poorly characterized due to difficulty in attributing species identities to biological collections. This research gap stems from an absence of type material and biological collections, also the confused diagnosis of *V. cruziana*. With the goal of improving systematic knowledge of the genus, we compiled information from historical records, horticulture and geography and assembled a morphological dataset using citizen science and specimens from herbaria and living collections. Finally, we generated genomic data from a subset of these specimens. Morphological and geographical observations suggest four putative species, three of which are supported by nuclear population genomic and plastid phylogenomic inferences. We propose these three confirmed entities as robust species, where two correspond to the currently recognized *V. amazonica* and *V. cruziana*, the third being new to science, which we describe, diagnose and name here as *V. boliviana* Magdalena and L. T. Sm. Importantly, we identify new morphological and molecular characters which serve to distinguish the species and underpin their delimitations. Our study demonstrates how combining different types of character data into a heuristic, total-evidence approach can enhance the reliability with which biological diversity of morphologically challenging groups can be identified, documented and further studied.

## Introduction

Reliably documenting plant diversity is necessary to protect and sustainably benefit from it. At the heart of this lie species concepts and the practical methods used to delimit taxa. Since Darwin first linked the phenomenon of speciation to that of evolution, systematic biologists have largely conceived of species mechanistically, equating them with separately evolving lineages equivalent to branches of the ‘Tree of Life’ (de Queiroz, 2007; Padial and De la Riva, 2021), with the logical consequence that the basis and process of species delimitation centres on assigning individuals to a phylogenetic lineage (de Queiroz, 1988, 1999, 2007; Mayo, in press). However, others argue that lineage divergence alone is not sufficient to delimit species (Freudenstein et al., 2017; Wells et al., 2021; Lavin and Pennington, in press). For example, Lavin and Pennington provide several examples in plants of mechanisms that yield paraphyletic species. Templeton in his ‘Cohesion Species Concept’ instead applies explicitly evolutionary criteria to define species as, “the most inclusive group of organisms having the potential for genetic and/or demographic exchangeability” (Templeton, 1989, p. 181). The heuristic approach, which is an extension of this idea, aims to reconcile the theory and practice of species delimitation (Wells et al., 2021). This proposes species as the outcome of their constituent individuals responding to similar suites of ecological and evolutionary forces in the same way and recognizes them in practice due to congruence in properties shaped by these forces. In addition, by advocating the application of multiple categories of data, we believe that this approach to delimiting species best reflects both the quality and the breadth of observations available (Monro, in press), whilst also overcoming the limitations of lineage-based approaches (Wells et al., 2021).

**FIGURE 1:**
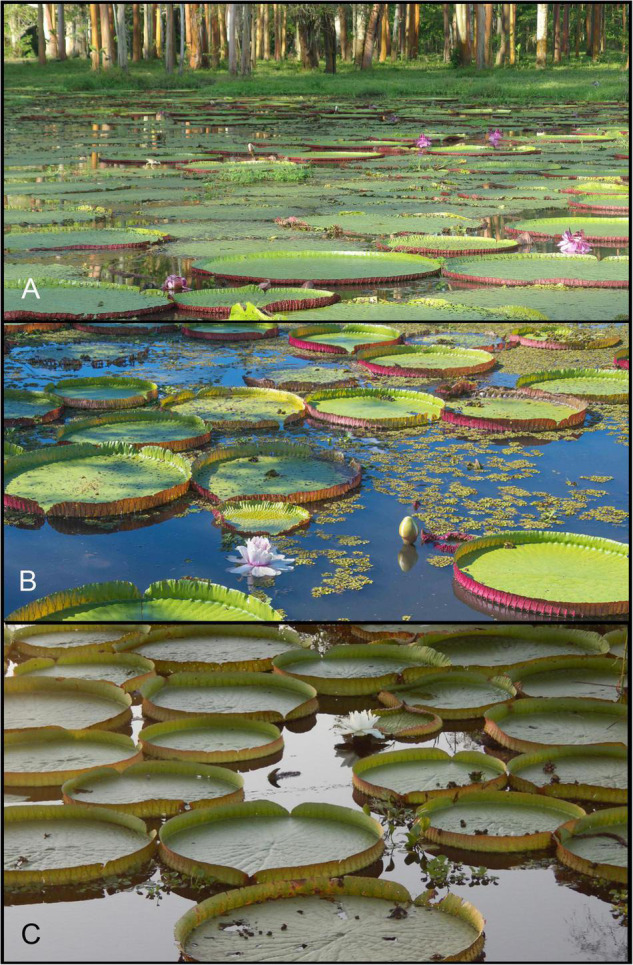
Wild populations of **(A)** *Victoria amazonica* (Peru, Loreto), **(B)** *V. boliviana* sp. nov. (Bolivia, Beni) and **(C)** *V. cruziana* (Argentina, Chaco). (Photo Credits: **(A)** Laurel Allen, iNaturalist ID 821386. **(B)** Carlos Magdalena. **(C)** Fernanda Alarcón, iNaturalist ID. 19516477).

Here we seek to apply a heuristic approach to the delimitation of species in the giant water lily genus *Victoria* Lindl. (Nymphaeaceae), a small and charismatic taxonomic group of short-lived perennial aquatic species distributed in the Amazonas and Chaco biogeographical regions of South America and famed for their enormous prickly leaves and massive blooms (Figure 1).

### Indigenous Names for Giant Water Lilies

Long before the description of the taxa by Poeppig (1832); Schomburgk (1837), and d’Orbigny (1840), *Victoria* was well-known to the Indigenous Peoples of South America, featuring in indigenous narratives (Prance and Arias, 1975) and with only partially documented cultural usages.

Local names for *V. amazonica* that have been recorded include ‘*auapé-yaponna,’* after auapé (*Jacana jacana*), a small bird often seen running on its leaves (Schultes, 1985; Box et al., 2022). *Victoria cruziana* has been called ‘*yrupé,’* ‘*yacare yrupé,’* or ‘*naanók lapotó*’ (‘*poncho del yacaré*’) (Crovetto, 2012; Scarpa and Rosso, 2014; Mereles et al., 2020).

### Assigning the Genus Name *Victoria* – In Search of Patronage

In 1832, after having been noted by several botanical explorers (”Haenke in 1801 and Bonpland in 1819,” and d’Orbigny in 1827; d’Orbigny, 1835, 1840), the German explorer and naturalist Eduard Friedrich Poeppig described a giant Water Lily, *Euryale amazonica*, that he had encountered in the environs of the Solimões river in Brazil that same year. Despite news of this discovery spreading quickly through Germany, it apparently reached neither Paris nor London and five years later the same species was described again, almost simultaneously, by the German botanist, Robert Schomburgk (Schomburgk, 1837) and the British botanist John Lindley (Lindley, 1837), under two different species epithets (*regina*, *regia*) and as a new genus (*Victoria*). Lindley’s species epithet prevailed in usage, possibly because of the great pomp with which it was delivered and its use to lobby a new monarch:

“It is therefore not less my duty …. in distinguishing your Majesty’s illustrious name, by far the most majestic species in the family of the Nymphs – one of the most noble productions of the Vegetable Kingdom – found in your Majesty’s South American dominions by a gentleman [Schomburgk] traveling under the auspices of your Majesty’s Government…” [Lindley, 1837].

The naming of the species was of great significance for the British scientific establishment as it came at a strategically important time, in the first months of the reign of Queen Victoria and at a time when several institutions were lobbying for royal patronage. Lindley’s opportunistic description of *Victoria regia* not only helped the Royal Geographical Society and the Horticultural Society of London (now Royal Horticultural Society) obtain patronage from Queen Victoria (Opitz, 2013), but also contributed to the decision not to close the Royal Botanic Gardens, Kew (Hooker, 1847).

According to Opitz (2013), it was also the first signal of the prominent status that science would come to play during her reign. The description of this species therefore had a much broader impact on botanical science in the British Victorian era, at a time when it was arguably at its most influential.

Given the fashion for greenhouses and cultivation of exotic plants, it is not surprising that the cultivation of *Victoria* in Europe and North America became a symbol of social status and horticultural achievement (Holway, 2013; Aniśko, 2014), with several Botanic Gardens even constructing dedicated greenhouses for the purpose. It could also be argued that it became a symbol of the British Empire, Paxton incorporating the structure of the leaf into the architecture of Crystal Palace (Aniśko, 2014).

### Further Nomenclatural History of *Victoria amazonica*

For most of the 19th and 20th centuries, the giant water lily from Amazonia was incorrectly known by the binomial *Victoria regia*. This was despite two earlier binomials for the taxon having priority, *Victoria* R. H. Schomb. for the genus name, and *amazonica* Poepp. for the species epithet.

Lindley had described *Victoria regia* [Victoria Regia: 3 (1837)] to accommodate material collected by Robert Hermann Schomburgk in Guyana. He did so amid some controversy (d’Orbigny, 1840; Opitz, 2013; Aniśko, 2014). Not only had Lindley prepared his manuscript in secrecy and against Schomburgk’s instruction, but he did so a month after Schomburgk had unwittingly published his in the Athenaeum (September 9 vs. October 16), thanks to a presentation on Schomburgk’s behalf by John Edward Gray to the Botanical Society (Gray, 1837). In all likelihood, Lindley’s secrecy reflected his appreciation of the role it could play in securing royal patronage and he wanted his name to have priority over John Edward Gray’s publication, whose preparation he was aware of. Lindley was, however, likely unaware of Poeppig’s earlier description [Froriep’s Not. Natur- Heilk. 25: 131 (1832)] but was soon made aware of it by a German correspondent, Weissenborn, who drew attention to the fact in the Magazine of Natural History (Weissenborn, 1837), a widely read British publication. On hearing of this earlier name, the epithet of which would have had priority over ‘*regia*,’ he did not either respond or correct the nomenclature and it was only 10 years later that the German botanist Johann Friedrich Klotzsch published the corrected combination, *V. amazonica* (1847). Lindley’s epithet, however, remained that most commonly applied to material of *V. amazonica* for over a century until Ghillean Prance’s clarification of the nomenclature of the species (Prance, 1974), after which time it was slowly replaced by *V. amazonica* (Poepp.) Klotzsch.

### A Second Giant Water Lily Species Described – *Victoria cruziana*, the Taxonomy of the Genus

*Victoria cruziana* Orb. was described as a second species by the French botanist Charles Henry Dessalines d’Orbigny [Ann. Sci. Nat., Bot., sér. 2, 13: 57 (1840)] based on material that he had collected in Corrientes, Argentina (considered Bolivia by d’Orbigny) in 1832. Since then, several additional names have been published, *Euryale bonplandia* Rojas Acosta [Cat. Hist. Nat. Corrientes: 151 (1897)], *Euryale policantha* Rojas Acosta [Cat. Hist. Nat. Corrientes: 65 (1897)], *Victoria trickeri* (Malme) Mutzek [based on *V. cruziana* f. *trickeri* Malme, Gartenwelt 29: 616 (1925)], all of which have subsequently been considered as synonyms of either *V. amazonica* or *V. cruziana*. In addition, two forms of *V. cruziana* were described by the Swedish botanist Gustaf Oskar Andersson Malme in 1907 [Acta Horti Bergiani 4(5): 12. 1907], *Victoria cruziana* f. *trickeri* Malme and *Victoria cruziana* f. *mattogrossensis* Malme.

Poeppig (1832) and Lindley (1837) disagreed over which genus the giant South American water lily should be assigned to, Poeppig assigning it to *Euryale* Salisb., a monotypic genus of large- leaved spiny water lilies from southern and eastern Asia, Lindley and Schomburgk considered it a distinct genus restricted to the neotropics. Both opinions are supported by molecular analyses which consistently recover species of *Victoria* and *Euryale* as sister to each other, either within (Löhne et al., 2007; Borsch et al., 2008), or sister to (Les et al., 1999; Borsch et al., 2008; Pellicer et al., 2013; Zhang et al., 2020) *Nymphaea*.

### Ethnobotanical Significance of *Victoria* in Its Natural Range

The large seeds of *V. cruziana* are consumed as a substitute for maize (Arbo et al., 2002; Mereles et al., 2020) and the rhizomes also have recorded usage as food (Scarpa, 2009). Similarly, amongst communities inhabiting the Paraguay river in the Pantanal region of Brazil, the seeds of *V. cruziana* f. *mattogrossensis* are ground with a pestle into a starch (Bortolotto et al., 2015). The petioles also have recorded usage as food (Kinnup and Lorenzi, 2014) and a juice obtained from the roots of *V. amazonica* is a source of natural black dye, used locally to color hair (Rosa-Osman et al., 2011). Medicinal uses of *V. amazonica* include wound treatment (Schultes, 1990), and *V. cruziana* has been recorded as an anti-inflammatory and a means for combating respiratory illnesses (Hurrell et al., 2016). The total cultural, spiritual and ethnobotanical knowledge of *Victoria* discovered by Indigenous Peoples is certainly more extensive, but poorly documented in the literature.

### Flower Morphology

**FIGURE 2:**
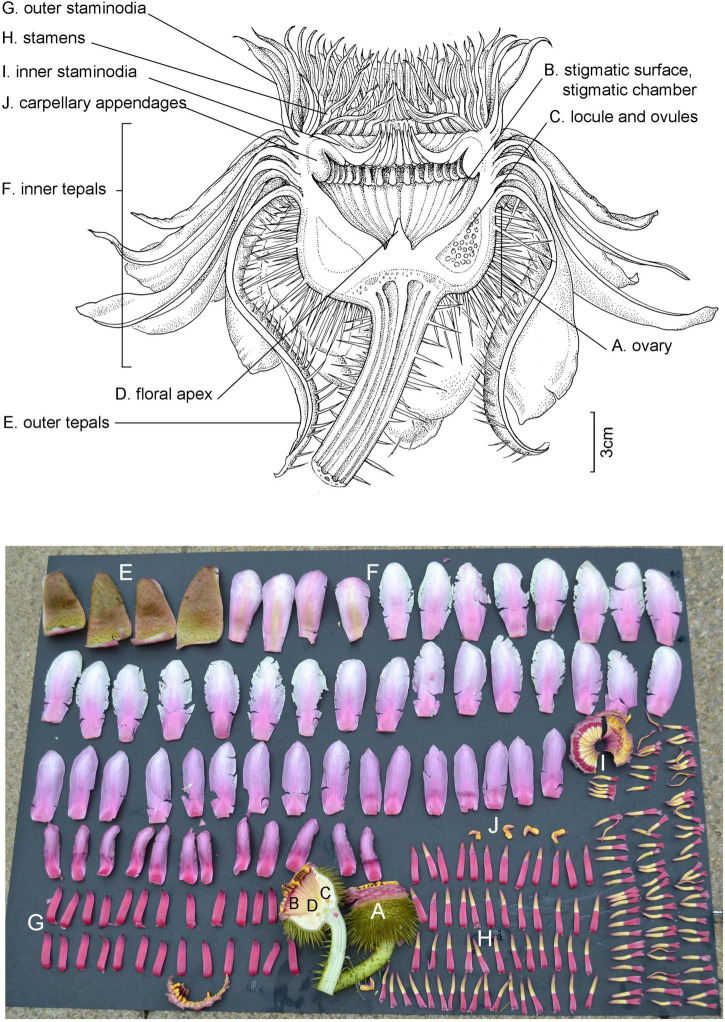
Flower morphology and terms, using a *Victoria amazonica* second-night flower in longitudinal section for reference (above) and a fully dissected flower (below). **(A)** Ovary, **(B)** stigmatic surface and stigmatic chamber, **(C)** locule and ovules, **(D)** floral apex, **(E)** outer tepals, **(F)** inner tepals, **(G)** outer staminodia, **(H)** stamens, **(I)** inner staminodia, **(J)** carpellary appendages. Illustration and photo: Lucy T. Smith.

**FIGURE 3:**
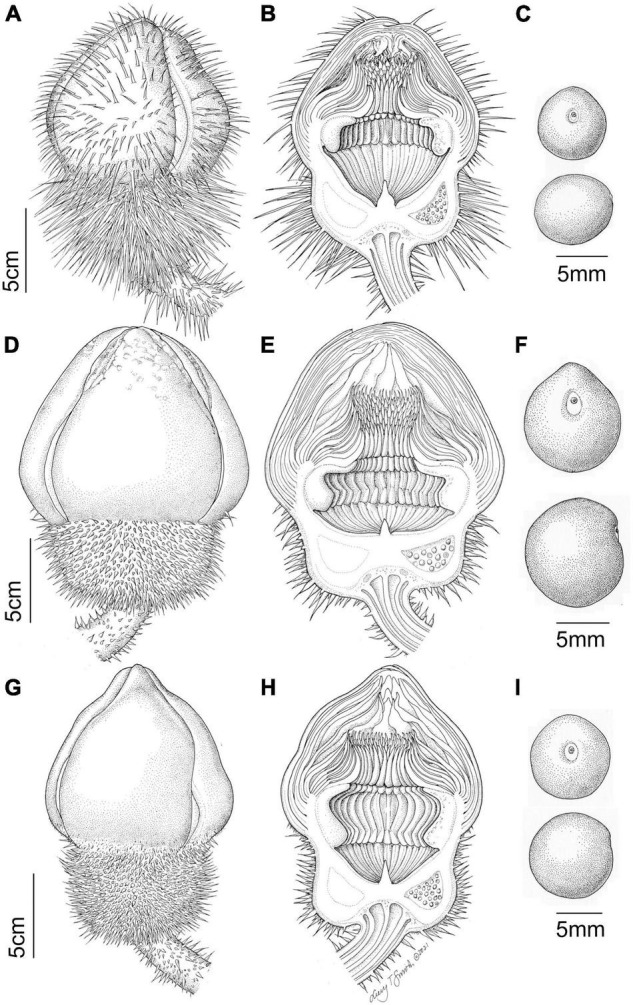
Line drawing comparing flower and seed morphology in: *Victoria amazonica* **(A–C)**, *V. boliviana* sp. nov. **(D–F)**, and *V. cruziana* **(G–I)**; flower bud whole and in longitudinal section showing abaxial surface of outer tepals, bud profile, stigmatic chamber, carpellary appendage and prickle morphology; seeds. (cultivated at RBG Kew) Illustration: Lucy T Smith.

**FIGURE 4:**
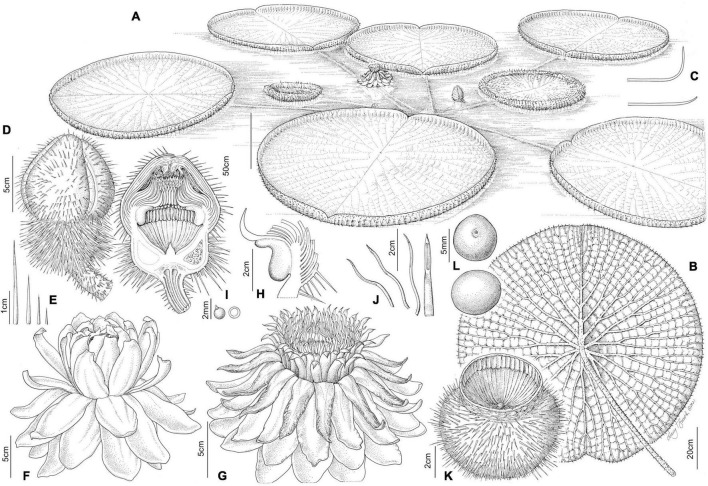
*Victoria amazonica* **(A)** habit, **(B)** abaxial leaf, **(C)** leaf rim profiles, **(D)** bud, whole and LS, **(E)** flower prickles, **(F)** first night flower, **(G)** second night flower, **(H)** carpellary appendages and tepal, staminode attachments; **(I)** ovule, whole and LS, **(J)** stamens, **(K)** fruit, **(L)** seed. (All from material cultivated at RBG Kew). Illustration: Lucy T. Smith.

**FIGURE 5:**
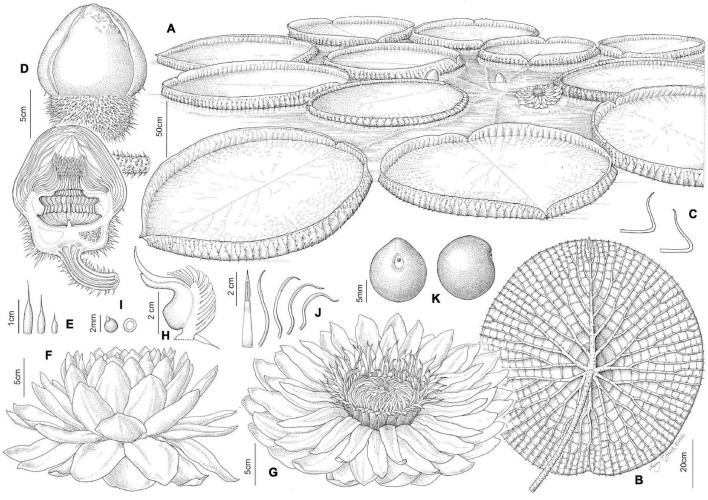
*Victoria boliviana* sp. nov. **(A)** habit, **(B)** abaxial leaf, **(C)** leaf rim profiles, **(D)** bud, whole and LS, **(E)** flower prickles, **(F)** first night flower, **(G)** second night flower, **(H)** carpellary appendages and tepal, staminode attachments; **(I)** ovule, whole and LS, **(J)** stamens, **(K)** seed. (All from material cultivated at RBG Kew). Illustration: Lucy T. Smith.

**FIGURE 6:**
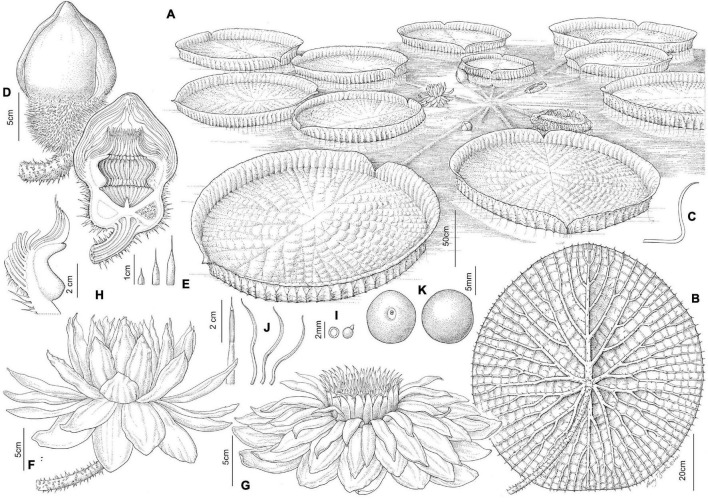
*Victoria cruziana* **(A)** habit, **(B)** abaxial leaf, **(C)** leaf rim profiles, **(D)** bud, whole and LS, **(E)** flower prickles, **(F)** first night flower, **(G)** second night flower, **(H)** carpellary appendages and tepal, staminode attachments; **(I)** ovule, whole and LS, **(J)** stamens, **(K)** seed. (**A–E, G–K**, cultivated at RBG Kew, **F** cult. Denver Botanic Gardens). Illustration: Lucy T. Smith.

Whilst the floral morphology of *Victoria* (Figures 2, 3, 4–6 and Table 1) has been well described, there has been no consensus in the terminology applied. The flowers have an inferior ovary, comprising 25–40 radially arranged syncarpous carpels (Figure 2A). The upper part of the ovary is a concave, papillose stigmatic surface, which is divided into raised segments (Figure 2B). Each segment corresponds to the roof of the locule below and bears a dorsal longitudinal slit through which pollen reaches the locules. Each locule contains 8–28 ovules, attached parietally to both sides of the locule’s walls (Figure 2C). At the center of the stigmatic surface is a column of residual stelar tissue, also known as the floral apex (Schneider, 1976; Figure 2D). There has been some inconsistency in the description of the perianth segments. Following Warner et al. (2008), we are referring to all perianth segments as tepals. From the apex of the ovary’s external rim, four rigid fleshy outer tepals arise (Figure 2E; Warner et al., 2008). From the tepals, helically arranged series of petaloid inner tepals follow (Figure 2F). Adjacent to these, and moving inwards, rigid, thick, outer staminodia arise, which are presented in a whorl-like arrangement of one or two whorls (Figure 2G). In bud and on the first night opening, the outer staminodia (along with the stamens and inner staminodia which follow) are sigmoid, and arch strongly over the stigmatic surface (Figures 3B,E,H). After these are sigmoid stamens (Figure 2H), borne in two or three whorls, pressed tightly against the outer staminodia in bud and during the first night of opening. The stamens are followed by sigmoid inner staminodia in one or two whorls (Figure 2I). The inner staminodia (previously classed as paracarpels: Prance and Arias, 1975) are partially adnate to the upper portion of the carpellary appendages, which lie beneath them. These L- shaped carpellary appendages (Figure 2J) are aligned with the locules below, and correspond with them in number. The lower portions of the carpellary appendages are abaxially fused to the rim-like extension of the stigmatic surface which is also fused with the basal tissue of the inner tepal bases.

**TABLE 1:** Source of contrasting morphological characters and their states from herbarium, horticultural, or field image data.

Character	Herbarium specimens	iNaturalist	Kew cultivated
(1) Leaf blade color adaxial (green, bronze, maroon)		X	X
(2) Leaf blade color abaxial (green, dark green/blue, yellow, maroon)		X	X
(3) Leaf rim shape (absent/present. Curving gently or perpendicular/recurved over adaxial surface at base/sigmoid & flared at top)		X	X
(4) Leaf rim height proportionate to length (absent, low, moderate, high, and as percentage of length)		X	X
(5) Leaf rim color abaxial (pale green/“white,” green, green tinged pale maroon, deep maroon)		X	X
(6) Hairs on abaxial leaf (size and number of segments, total hair length, density)	X		
(7) Shape of bud (apex convex/concave toward tip)	X	X	X
(8) Prickles on outer tepals abaxial, presence or absence	X	X	X
(9) Prickles on outer tepals abaxial, distribution (covering or partially covering, where on outer tepal)	X	X	X
(10) Prickles on outer tepals abaxial, number	X	X	X
(11) Prickles on outer tepals abaxial, spacing (regular or irregular)	X	X	X
(12) Prickles on outer tepals abaxial, shape (smoothy tapering to sharp point or abruptly tapering to sharp point)	X	X	X
(13) Prickles on outer tepals abaxial, range of sizes and actual sizes	X	X	X
(14) Hairs on outer tepals (visible to naked eye or not)	X	X	X
(15) Prickles on ovary (present on all species), shape (smoothly tapering to sharp point or abruptly tapering to sharp point)	X	X	X
(16) Prickles on ovary (present on all species), number of sizes, and actual sizes	X		X
(17) Color of innermost tepals in bud and on first-night opening (white/dark or maroon)	X	X	X
(18) Shape and depth of stigmatic chamber (deep/shallow, oblong/triangular/rounded)		X	X
(19) Color of open first-night flower, inner tepals (white, white with maroon innermost tepals)		X	X
(20) Texture of inner tepals on first-night opening (smooth/crinkled)	X	X	X
(21) Color of open second-night flower inner tepals (white, pale pink, dark pink, maroon)	X	X	X
(22) Carpellary appendages, shape of lower part (fixed, arising flat from surface/free, curved)	X	X	X
(23) Carpellary appendages, size of upper part and lower parts and their relationship (smaller than/greater than/equal to equal to)	X	X	X
(24) Size of ovules	X		X
(25) Number of ovules per locule			X
(26) Number of seeds per fruit			X
(27) Seed size	X		X
(28) Seed shape (globose, ellipsoid, raphe distinct or not)	X		X

The cavity enclosed by these parts is referred to as the stigmatic chamber (Figure 2B). In bud and during the first night of flowering, the apices of the outer staminodia, stamens and inner staminodia are pressed tightly into each other to form an entrance tunnel (Figures 3B,E,H). This tunnel remains intact in bud and during the first night of opening, providing entry to the beetle pollinator, but is absent on the second night of opening, when all the outer staminodia, and most of the stamens, reflex to varying degrees (Figure 2). The inner staminodia, however, do not reflex, but instead fall further downwards, thus blocking the entrance to the stigmatic chamber. Flower size, the number of locules and the number of all parts (apart from the outer tepals, which always number four) are variable both between individual plants and on different flowers produced by the same plant.

### Pollination and Dispersal Biology

*Victoria* flower buds develop underwater and emerge above the surface when ready to bloom. Each flower opens over two consecutive nights, changing form and color dramatically in- between. These form and color changes reflect their role in pollination, which is to trap pollinating beetles (Schomburgk, 1837; Prance and Arias, 1975) of the Cyclocephalini tribe (Scarabaeidae) (Prance and Arias, 1975). Floral morphology of the fossil *Microvictoria*, suggests that this mode of pollination was already established in the Cretaceous (Gandolfo et al., 2004). The carpellary appendages are believed to produce scent attractants and nutritional rewards (Prance and Arias, 1975; Zini et al., 2019) and with the warming of the flower (thermogenesis) at night (Planchon, 1850, 1851; Knoch, 1899; Decker, 1936; Prance and Arias, 1975; Lamprecht et al., 2002) serve as both an attractant and stimulant for the pollinators (Seymour and Mathews, 2006). The stigmatic surface remains receptive for the two nights during which the flower blooms (Archangeli, 1908). Despite the above investment in cross-pollination, Prance and Arias (1975) demonstrated that self-pollinated flowers were still capable of setting seed. Furthermore, seed produced in cultivation as a result of selfing is viable (Magdalena, personal observation). There have been few published pollination studies of *V. cruziana* and only one record of the putative new species from the Mamoré river basin in Bolivia (Magdalena, personal observation). After pollination, the fruit forms below the water surface (Prance and Arias, 1975). The seeds are covered by a mucilaginous tissue that has been proposed to represent an aril, are buoyant for a few days and released as the fruit decomposes (Prance and Arias, 1975). Prance and Arias (1975) suggest that seeds of *V. amazonica*, produced at the end of the wet season, are dispersed over long distances because of the annual flooding of much of its habitat and this may also be the case for *V. cruziana*. Although there is no evidence of endochory in the genus, this should not be excluded as the dispersal biology of the species remains very poorly studied.

### Ecology and Biogeography

*Victoria* occurs in white-water and occasionally black-water (Byrne, 2008) leas and igapos of the Amazonian and Paraná river basins (Rosa-Osman et al., 2011) at depths of up to 5.25 m (*V. amazonica*, Prance and Arias, 1975). *Victoria* spp. are commonly classified as annuals but Prance and Arias (1975) propose that, in the case of *V. amazonica* at least, this life cycle likely reflects a constraint posed by the dramatic changes in water level associated with flooding and drought, which characterize the wet and dry seasons across the range of this species. In a stable horticultural environment, *V. amazonica* and *Victoria* ‘Longwood’ hybrids can thrive for several years. They should, therefore, be considered to be short-lived perennial species. The seeds of *V. amazonica* are desiccation intolerant (Rosa-Osman et al., 2011). Seedlings develop very quickly in the river mud forming mature plants in three to five months. Development is quicker in *Victoria cruziana* compared to *V. amazonica* (Kit Knotts et al., personal observation). This is possibly a reflection of the shorter and more predictable growing season in the temperate biome of *V. cruziana.* Senescence is triggered by the detachment of the rhizome from the riverbed, or desiccation as the river level drops (Aniśko, 2014).

### Taxonomic Challenges

Taxonomic treatment of *Victoria* has been hampered by several factors. Foremost is the fact that the type collections of the two currently recognized species (World Checklist of Vascular Plants, 2022) have been lost or destroyed, making it challenging to unambiguously name material and thus delimit species. Poeppig’s collection(s) at Naturhistorisches Museum Wien and University of Leipzig were likely destroyed during WW2 and d’Orbigny’s s spirit collection disappeared from the Paris museum for reasons unknown. In addition, d’Orbigny diagnosed *V. cruziana* against material of *Victoria* morphotype *‘boliviana’* that he had mistakenly considered to be *V. amazonica* and in doing so established misleading species-limits at a time when very few collections were available for study. The above issues were compounded by the fact that *Victoria* is notoriously difficult to make herbarium specimens from, being big, fleshy, covered in prickles and prone to rotting in the dryer. This likely explains why there are relatively few herbarium specimens of wild collected material: only 97 of *V. amazonica* and 18 of *V. cruziana* (The Global Biodiversity Information Facility [GBIF], 2022), despite the species’ broad distributions.

These reasons could explain why the genus attracted relatively little taxonomic attention in the 20th Century, during which the most notable contributions were made by Malme (1907) and Prance and Arias (1975). In the 21st Century, renewed interest was, associated with research for the Flora of Brazil. Notably Pellegrini’s treatment for the Flora of Brazil (2020) that recognizes the two species, and de Lima et al. (2021), which recognize a single variable species (*V. amazonica*).

Significantly, much of our knowledge regarding the natural history of *Victoria* has been obtained from material growing in cultivation and compiled by horticulturalists in the additional literature or worldwide web^1^ and it was observations by C. Magdalena which first suggested the existence of additional species and the need for a re-evaluation of the genus.

The aims of this study were to (1) revisit species delimitation in *Victoria* and to do so through an iterative process, using morphological observations to establish species hypotheses and suites of observations to test these, and in doing so (2) reveal the principle evolutionary lineages, their age, permeability and biogeography, (3) identify diagnostic morphological and DNA sequence characters for the species, and use the above to (4) revise the nomenclature, descriptions, distribution, and conservation status of the species.

## Materials and Methods

We applied a heuristic species concept (Wells et al., 2021) in which morphological, field and horticultural observations were used iteratively to develop initial hypotheses of species limits that we further tested using rigorous phylogenomic and population genomic analyses. This study was an Anglo-Bolivian collaboration instigated simultaneously by both parties with the goal of conducting research into *Victoria* in an equitable manner (McAlvay et al., 2021).

### Morphological Evaluation of Putative Species

#### Taxon Sampling

Morphological observations were made from as many gatherings and images of plants as possible. We examined 110 sheets of 58 herbarium collections both physically and digitally (via Reflora and JSTOR Plants) held at BM, COR, HGCS, IAN, JBRJ, K, LPB, MO, NY, P, SI, SPF, UBCB, and US (abbreviations according to Thiers, 2016). These were supplemented with 175 ‘research grade’ geolocated field images from iNaturalist, images publicly available on Instagram, Facebook, Googlesearch, and living collections at K. A subset of the herbarium specimens was used as the source of DNA for the genomic analyses (Supplementary Table 1). This subset was designed with the aim of including representatives of at least three gatherings per morphotype and principal river basin (Amazon, Essequibo, Paraná), including those from or close to the type localities of *Victoria amazonica*, *V. cruziana* and *V. cruziana* f. *mattogrossensis*. These included a type of element of *Victoria cruziana* (d’Orbigny s.n., P02048598).

#### Morphological Observations

The selection of the morphological characters recorded was based on our field and horticultural observations and experience of examining herbarium material, characters used in previous studies (notably Malme, 1907) and those which could be observed from herbarium, living collections or research quality geo-referenced photographic images on iNaturalist. The herbarium and iNaturalist datasets complemented each other well since each favored a particular set of characters (Table 1). For example, herbarium collections were a good source of observations of prickle morphology, number and distribution, carpellary appendages, tepal pigmentation, trichomes, seed size and shape. iNaturalist images were a better source of observations of gross morphological characters such as the shape and height of leaf rims and flower color. Where close-up photos were included in iNaturalist records, it was also possible to examine prickle morphology, number, and distribution.

Twenty-seven morphological characters were documented for living collections at RBG Kew (Table 1), encompassing observations of every part of the plant. These were also used to calculate rim heights as a proportion of leaf length (Tables 1, 2). Nineteen morphological characters were documented for herbarium specimens (Table 1 and Supplementary Table 4), encompassing: leaf indument (rim, lamina), presence or absence and distribution of prickles on outer tepals and their morphology, number and distribution, tepal number, carpellary appendage size and shape, ovary indument and prickle morphology, fruit prickle morphology, color of innermost tepals in bud, and seed size. Due to the size of *Victoria* leaves, the size of a herbarium sheet and the flattening of leaf structures, specimens were not a source of observations on leaf size or rim height. Twenty-one morphological observations were documented from iNaturalist images (Table 1 and Supplementary Table 5) encompassing adaxial and abaxial leaf color, rim shape and color, bud, first- and second-night flower color, and occasionally floral characters such as the distribution, morphology and number of outer tepal prickles and the color of innermost tepals in bud.

**TABLE 2:** Morphological character states used to delimit morphospecies of *Victoria*.

Character	*V. amazonica*	*V. boliviana*	*V. cruziana*	*V. c.* f. *mattogrossensis taxon incertum*
Rim shape of mature leaf in cross-section	Curved at base, perpendicular Not flared at apex	Strongly recurved over adaxial surface Curling inwards or flared at apex	Recurved over adaxial surface at base Flared at apex, sigmoid	Strongly recurved over adaxial surface Curling inwards or flared at apex
Rim height	Absent, or low to moderate, 4–7% leaf length, higher only in congested areas	Moderate, 5–7% leaf length	Moderate to high, 8–10% leaf length	Moderate, unknown % leaf length
Rim color	Maroon, occasionally green	Maroon/red, or pale green	Green, or tinged pale maroon	Maroon/red
Leaf trichome length (where present)*	0.3 – 12 mm	1.2 – 3 mm	1 – 3 mm	?
Leaf trichome segment number*	3 – 12	6 –15	10-15	?
Bud shape*	Convex at apex	Convex at apex	Concave just before apex	Convex at apex
Ovary prickles shape	Smoothly tapering to a point	Abruptly tapering tapering to a point	Abruptly tapering to a point	Abruptly tapering to a point
Ovary prickle size (dried)	2 – 21 mm	1 – 10 mm	1 – 22 mm	2 – 15 mm
Ovary trichome length (where present)*	0.1 – 0.4 mm	NA	0.1 – 12 mm	??
Ovule number*	20–28	8–14	20–25	??
Ovule size (fresh) (dried)	1.5 mm 0.5 – 1.5 mm	2–2.5 mm 2–2.5 mm	1.5 – 1.8 mm 1.2 —1.5 mm	– –
Abaxial outer tepal color	brown/maroon	Green or maroon	Green or maroon	Green or maroon
Abaxial outer tepal prickles	Present	Absent or present	Absent or present	Present
Abaxial outer tepal prickle number*	55 – 330	0 – 10	0 – 100	300 – 1000+
Abaxial outer tepal prickle shape	Smoothly tapering to a point	Abruptly tapering to a point	Abruptly tapering to a point	Abruptly tapering to a point
Abaxial outer tepal prickle distribution*	Covering entire surface	Covering entire surface	Covering basal third only	Covering entire surface
Abaxial outer tepal prickle arrangement	Regularly to irregularly spaced	Irregularly spaced	Regularly to irregularly spaced	Regularly spaced
Abaxial outer tepal prickle (dried)	1 – 14 mm Two – three sizes, two sizes predominant	2.5 – 5 mm Three sizes	1 –7 mm One – four sizes	1 – 4 mm Three sizes, two predominant
Abaxial outer tepal trichome length (where present)*	0.1–0.2 mm	NA	0.1 – 1 mm	??
Color of innermost tepals in bud and on first night opening*	Dark red/maroon	White	White	White
Proportionate lengths of upper and lower arms of L-shaped carpellary appendages*	Upper part equal to or shorter than lower part	Upper part greater than lower part	Upper part smaller than lower part	Upper part smaller than lower part
Shape of lower arm carpellary appendage at base*	Rounded, hanging free, auriculate	Straight, partly free	Straight, attached	Straight, partly free
Seed shape	Ellipsoid	Globose	Globose	Globose
Seed raphe*	Raphe faintly visible	Raphe prominent	Raphe faintly visible	Raphe prominent
Seed dimensions*	7–8 × 9–10 mm	12–13 × 16–17 mm	8–9 × 9–10 mm	6–10 × ca 10 mm

* denotes novel character in the table.

#### Geographical Observations

Geographical observations were used both to map the distribution of putative *Victoria* species and to undertake extinction risk assessments. Localities were taken from the labels of herbarium collections and the metadata of iNaturalist records and recorded as decimal coordinates (Supplementary Table 2).

For collections where there was no coordinate data but where precise locality information was given, GoogleEarth was used to estimate latitudes and longitudes. We also reviewed social media accounts of water lily enthusiasts using tags for *Victoria* (Facebook, Instagram, Googlesearch). Whilst useful, social media posts were not considered as a reliable source of geographical coordinates for Victoria populations; unlike iNaturalist, these platforms do not curate spatial data and posts can be removed or edited at any time. Social media posts were deemed unsuitable for use in the calculation of extinction threat assessments but valuable in providing an indication of hitherto undocumented populations that could then be confirmed by directly contacting the posters and confirming the locality using Googlearth. Because *Victoria* grows in open stretches of riverbank, it is possible to recognize *Victoria* populations in Google Earth images, due to their distinctive leaf outline and size, and contrast with a body of water.

#### Extinction Risk Assessments

Extinction risk assessments were undertaken using IUCN Red List Categories and Criteria of Threatened Species (Hereafter IUCN Red List) version 3.1 (IUCN, 2001; IUCN Standards and Petitions Committee, 2019). Calculations of the extent of occurrence (EOO) and area of occupancy (AOO) were undertaken using the online conservation assessment tool GeoCAT (Bachman et al., 2011). The estimated AOO was calculated using a cell width of 2 km as recommended by IUCN and the estimated EOO was calculated based on the minimum convex polygon (IUCN Standards and Petitions Committee, 2019). Due to the availability of a relatively small number of herbarium specimens we calculated and contrasted a maximum and minimum range. The maximum range included potential habitat within the range, whilst the minimum range was limited to confirmed observations, either from herbarium specimens or iNaturalist posts. Unverified images were defined as “presence uncertain” and excluded from the minimum estimate but included for the maximum range. The extinction risk assessments undertaken here will be uploaded to the IUCN Species Information Service (SIS) in 2022 after completion of the official peer reviewed process and official submission to the IUCN Red List.

### Genomic Evaluation of Putative Species

#### Taxon Sampling

Leaf tissue was sampled from 18 specimens obtained from both herbarium collections (*n* = 12, K, MO, HGCS, and P) and living collections [*n* = 6, Adelaide Botanical Garden, Australia (AD), K, Santa Cruz Botanic Garden, Bolivia, Royal Botanic Garden, Kew (K)]. Samples from the living collections included three individuals of the putative new species (seeds from Santa Cruz Botanic Garden) as well as an outgroup [*Nymphaea ampla* (Salisb.) DC.] (Supplementary Table 1). Samples from living collections and the field are hereafter referred to as “fresh” samples. These tissue samples were stored in silica gel prior to DNA extraction. All specimens were used in compliance with loan agreements of the source biological collections (K, MO, P, and HGCS).

#### DNA Extraction, Library Preparation and Sequencing

A total of 20–40 mg leaf tissue was weighed out and pulverized using a SPEX^®^ sample prep tissue homogenizer (SPEX Inc, Metuchen, NJ, United States). DNA was extracted using CTAB and isopropanol (Doyle and Doyle, 1987) and cleaned using a 2x ratio of AMPure XP beads (Beckman Coulter, Brea, CA, United States). DNA libraries were prepared using NEB Next Ultra II Library Prep Kits according to the manufacturer’s protocol (with half volume reactions) and with NEBNext Multiplex Oligos for Illumina (New England Biolabs, Ipswich, MA, United States) amplified with 9–11 PCR cycles (fresh samples) or 11–15 PCR cycles (herbarium samples). Yield and fragment size distribution were estimated using a Quantus fluorometer (Promega, Madison, WI, United States) and a 4200 TapeStation system (Agilent Technologies, Santa Clara, CA, United States) respectively. Sequencing of DNA libraries was carried out on an Illumina NovoSeq platform with a paired end 150 bp configuration, by GeneWiz^®^ (South Plainfield, NJ, United States).

#### Generating Transcriptome-Based *Victoria* Nuclear Reference Reads

Since no genome assembly is currently available for genus *Victoria*, we used transcriptome reads to create a *Victoria* genomic reference for read mapping. The published transcriptomic data was obtained from a *V. cruziana* sample (SRX6884057) (Zhang et al., 2020) sequenced on an Illumina platform. We trimmed adaptor sequence from the reads using Trimmomatic v. 0.39 (Bolger et al., 2014), with sliding window trimming, cutting once the average quality across 4 bases fell below a PHRED score of 20 and requiring a minimum length of 30 bp. We then carried out a *de novo* assembly of the reads using Trinity v.2.8.5 (Grabherr et al., 2011) with default settings. We estimated transcript abundance (where a minimum threshold value acts as a proxy for ‘real’ genes) using the alignment- free method *salmon*. Subsequently, we filtered the raw assembly for transcripts of low expression with a normalized TPM (transcripts per million) matrix, where transcripts with an expression level < 1 TPM for any given sample (an expression level of at least one could be of biological relevance) and retained only the most expressed isoform of each transcript. This resulted in retention of 74,088/152,932 (48.45%) transcripts. We then used CD-HIT (Fu et al., 2012) to cluster all of the transcript sequences and retain only one read from any clusters of similar sequences, where the identity threshold was set to 0.95. This step filtered out 172 (<0.4%) of transcripts. Finally, we removed all transcripts of length < 350 bp (an assumed minimum insert size, given that Illumina sequencing of 150 bp paired-end reads was conducted). This resulted in a *Victoria* genomic reference set of 38,703 transcript sequences. To ensure that our population genetic analyses were exclusively derived from nuclear SNPs and not from organellar or fungal/bacterial DNA sequences, we conducted a remote blast search of the newly assembled transcriptome against the entire NCBI nucleotide database, using the *blastn* software of the NCBI tools (Camacho et al., 2009), an e-search value of 0.001 and keeping a maximum of five hits per queried sequence. We discovered that 820 (2.1%) and 612 (1.6%) of the transcripts respectively matched chloroplast and mitochondrial sequences, and thus were removed from subsequent analysis. In addition, ∼0.85% of the total proportion of blasted transcriptomes matched bacterial or fungal DNA sequences, indicating that the presence of contaminant reads in the assembled transcriptome is negligible. Our filtered *Victoria* reference set derived from the assembly totaled 37,470 transcripts. Finally, to assess completeness of the *de novo* assembly, we used BUSCO v. 5.3.2 (Simão et al., 2015), applying the lineage dataset chlorophyta_odb10 [constituting 16 genomes and 1519 benchmarking universal single-copy ortholog (BUSCO) genes].

#### Processing of High-Throughput DNA Sequence Data and Alignment to Transcripts

We trimmed the raw read data using AdapterRemoval v2.3.2 (Schubert et al., 2016) with the ‘collapse’ option to maximize retention of shorter reads, a consideration based on our dataset having a large proportion of herbarium specimens (Latorre et al., 2020). We aligned the trimmed reads to the transcriptomic reference set of reads using bwa v 0.7.17 (Li and Durbin, 2009), with the *mem* algorithm (suited to long reads and seeds alignments with exact matches) for the samples from fresh material and *aln* for the herbarium material (suited to short reads and allows for mismatches). We retained reads with a minimum mapping quality of 20 and of a minimum length of 25 bp (herbarium samples) and 30 bp (fresh samples) and removed PCR duplicates using the function *rmdup* of the software samtools v.1.7 (Li et al., 2009). Endogenous content (the proportion of *Victoria* DNA sequence compared to exogenous reads) was calculated by comparing totals of mapped reads (before PCR duplicate removal) to totals of trimmed reads. Mean sequencing depth was calculated along the entirety of the aligned sequence for each sample.

#### Population Genomic Analysis of Nuclear Data

Given the shallow phylogenomic scale under investigation in this study and due to the high prevalence of inter-specific hybridization characterizing the family *Nymphaeaceae* (Borsch et al., 2014; Robson et al., 2016), and the potential of inter-specific hybridization to interfere with inference of the species trees in flowering plant taxa (Pirie, 2015; Morales-Briones et al., 2021; Pérez-Escobar et al., 2021a,b), we applied a population genomic approach. We excluded the *Nymphaea* outgroup from this analysis, leaving our set of 16 *Victoria* samples. Due to the low average depth of sequencing afforded by our genome skimming approach (see *Results*), we estimated genotypes using a genotype likelihood (GL) method in ANGSD v.0.933 (Korneliussen et al., 2014). In this approach, genotype likelihoods were scored inferring major and minor alleles and retaining sites with *p*-value of at least 1e-6 and a minimum mapping quality of 30. We used these genotype likelihoods to carry out a principal component analysis (PCA) with PCAngsd (Meisner and Albrechtsen, 2018), with default settings and a maximum of 10,000 iterations. Due to the very small sample size, we chose a stringent threshold for genotype missingness across all samples – a maximum of 1 missing individual before rejection of the site (-*minInd* set to 15) and minor allele frequency (*maf*) thresholds of 0.1 and 0.2 were applied. Minor alleles can have a disproportionately large effect on population structure inference (Schmidt et al., 2021); singletons and doubletons will be very common given the very restricted sample size here, thus we would expect the results from the *maf* = 0.2 filtering run to more accurately represent the true genomic structure. The *maf* = 0.1 iteration was performed for comparison, as a dataset comprising more genotyped sites in total.

#### Phylogenomic Analysis of Plastid Data

For the construction of a plastid phylogeny, we utilized a published chloroplast genome, of *V. cruziana* (Gruenstaeudl et al., 2017) available on the NCBI repository (NC_035632) as a reference genome. We aligned our trimmed reads to this reference and filtered them using bwa v 0.7.17 (Li and Durbin, 2009), using the same settings as above (see section “*Processing of high-throughput DNA sequence data and alignment to transcripts”*). We then used ANGSD to generate pseudohaploid (where diploid genomic data is simplified into a single consensus sequence) consensus sequences from the aligned reads, setting a minimum sequencing depth threshold of 10 and a minimum base quality score of 30. Using the 15 samples in which genotyping completeness was > 99.8%, along with *Euryale ferox* as an outgroup, we computed the maximum likelihood (ML) tree using the software RAxML v.8.2.12 (Stamatakis, 2014), with a GTR substitution model, the GAMMA model of rate heterogeneity and 500 bootstrap replicates. The genome for *Euryale ferox*, the tropical Asian water lily (NC_037719.1) (He et al., 2018) was sourced from the NCBI repository.

#### Molecular Dating Analysis

To elucidate the absolute times of divergence amongst populations of *Victoria*, we relied on the implementation of molecular clocks and multispecies coalescent (MSC) models in the program StarBEAST2 v.2.5 (Ogilvie et al., 2017), on the same whole plastid genome data produced to compute a ML phylogeny (see section “*Phylogenomic analysis of plastid data”*). This approach enables the estimation of calibrated species trees using population sampling information while considering topological gene tree incongruence such as the one derived from incomplete lineage sorting (ILS) of gene flow (Ogilvie et al., 2017). One partition representing the entire whole chloroplast genome and a total of 158,992 sites (of which 390 were informative) was used as input, containing linear sequences of three individuals representing populations of *V. ‘boliviana’* morphotype, four individuals representing *V. cruziana*, eight individuals representing *V. amazonica*, one individual of *Euryale ferox* and one individual of *Nymphaea ampla*, the latter two employed as outgroups. Following the times of divergence obtained by Zhang et al. (2020) for Nymphaeales, to calibrate our plastid phylogeny, we relied on two secondary calibration points applied to: (a) the root of the tree representing the divergence of *Victoria* and *Euryale* from *N. ampla*, set to 75 Ma, (b) to the MRCA of *Euryale* and *Victoria*, set to 36 Ma; both secondary calibration points were set to a normal prior distribution and a standard deviation of 1. We modelled the substitution rates with a GTR substitution model and rate heterogeneity among sites with a four-categories Gamma distribution in conjunction with a relaxed log-normal molecular clock. The molecular clock was informed using a uniform prior distribution for the mean rate, ranging from 1.0e-5 to 0.001, which represents a range of plastid substitution rates reported for different land plant lineages (Gaut et al., 1992). A ploidy level of “1” (option “Y or mitochondrial”) was indicated in the program, as recommended for plastid datasets (Drummond and Bouckaert, 2015). Lastly, a coalescent constant population tree model with a mean population size of 1.0 and a non-informative prior of 1/X was chosen, following (Drummond and Bouckaert, 2015) whenever a mixture of population-level sampling is involved. We executed 100 million generations in StarBEAST2, sampling every 5000 states and ensuring that all parameters reached convergence as evidenced by effective sample sizes > 200.

#### Comparative Genomics of *Victoria* Plastomes

To further investigate the genomic properties of the proposed new species (specifically: consistent variation in the form of point mutations, indels or structural variation), we *de novo* assembled the plastid genomes of *V. cruziana* (*n* = 2), *V. amazonica* (*n* = 1), and *V. ‘boliviana’* morphotype (*n* = 2) and created whole genome alignments. We took the raw reads from samples: NPNY23 (*V. cruziana*), NPNY21 (*V. cruziana*), NPNY14 (*V. amazonica*), NPNY24 (*V. ‘boliviana’* morphotype), NPNY26 (*V. ‘boliviana’* morphotype), trimmed these using stringent settings in Trimmomatic v.0.39; retaining only reads at least 50 bp long and removing bases with a Phred quality score below 30. These trimmed reads were the raw material for plastome assembly we subsequently performed with GetOrganelle (Jin et al., 2020), using default settings. The resulting complete genomes were aligned using Mauve v.2.3.1 (Darling et al., 2004), as implemented in the platform Geneious v.8.1.9 (Kearse et al., 2012). In this alignment, apart from the assembled genomes, we included plastid genomes from the NCBI repository: *V. cruziana* (NC_035632) as well as the plastid genome of *Euryale ferox*. Whole genome alignment was conducted using the mauveAligner algorithm with the following options: ‘full alignment,’ ‘extend local collinear blocks (LCB)’ and ‘automatically calculate minimum LCB score.’ Genomes were functionally annotated using the software GeSeq (Tillich et al., 2017) of the Chlorobox toolkit^2^ and the following parameters: a protein search identity value of 25, rRNA, tRNA and DNA search identity of 85, and the annotated plastid genomes of *E. ferox* (NC_037719.1) and *V. cruziana* as reference (NC_035632). The resulting annotated LCBs were scanned manually to detect indels and point mutations unique to the *V. ‘boliviana’* morphotype sequences. Finally, each LCB was analyzed in DnaSP v.6 (Rozas et al., 2017) to compute the variant sites between the three *Victoria* species, parsimony-informative sites and point mutations unique to *V. ‘boliviana’* morphotype.

#### Genome Size Estimation

Nuclear DNA contents were estimated by propidium iodide flow cytometry using fresh leaf material. Around 1 cm^2^ matured leaf tissue from the specimen was co-chopped with the internal standard [*Petroselinum crispum* (Mill) Nyman ex A. E. Hill ‘Champion Moss Curled,’ 1C = 2171.16 Mb (Obermayer et al., 2002)] using a new razor blade in 1 ml of General Purpose Buffer supplemented with 3% PVP (GPB) (Loureiro et al., 2007). A further 1 ml of GPB was added to the sample and the contents gently mixed in the petri dish. The sample was then passed through a 30 μm nylon filter. The homogenate was stained with 100 μl propidium iodide (1 mg/ml) and incubated on ice for 10 min. Two samples were prepared from the same individual and three replicates of each were run, recording up to 1,000 nuclei per fluorescence peak using a Sysmex CyFlow Space (Sysmex Europe GmbH, Norderstedt, Germany) flow cytometer fitted with a 100 mW green solid state laser.

The resulting histograms were analyzed with the WindowsTM-based FlowMax software (v. 2.9 2014, Sysmex GmbH) and the average of each sample was used to estimate genome size.

#### Chromosome Count

A chromosome count was obtained from a single individual of *V. ‘boliviana’* morphotype. growing in the RBG Kew Living Collection (accession number x2018-659) using the conventional root squash method to observe mitotic chromosomes (Pellicer et al., 2007). Briefly, actively growing root tip meristems were collected and pre-treated with aqueous colchicine [0.05% (v/v)] for 4 h, then transferred to freshly made Carnoy’s fixative [3:1 (v/v) absolute ethanol and acetic acid] for 24 h at ∼21°C. Root tips were then transferred to 70% (v/v) ethanol and stored at –20°C until used. Before squashing, root tips were hydrolyzed in 1M HCl at 65°C for 8 min, transferred to 2% aceto-orcein and stored at 4°C overnight. Each root tip was placed on a microscope slide and squashed under a 22 × 30 coverslip in a drop of 4.5% acetic acid and analyzed under a Leitz Laborlux D phase-contrast microscope (Ernst Leitz Wetzlar GMBH, Germany).

## Results

### Morphological Observations

**FIGURE 7:**
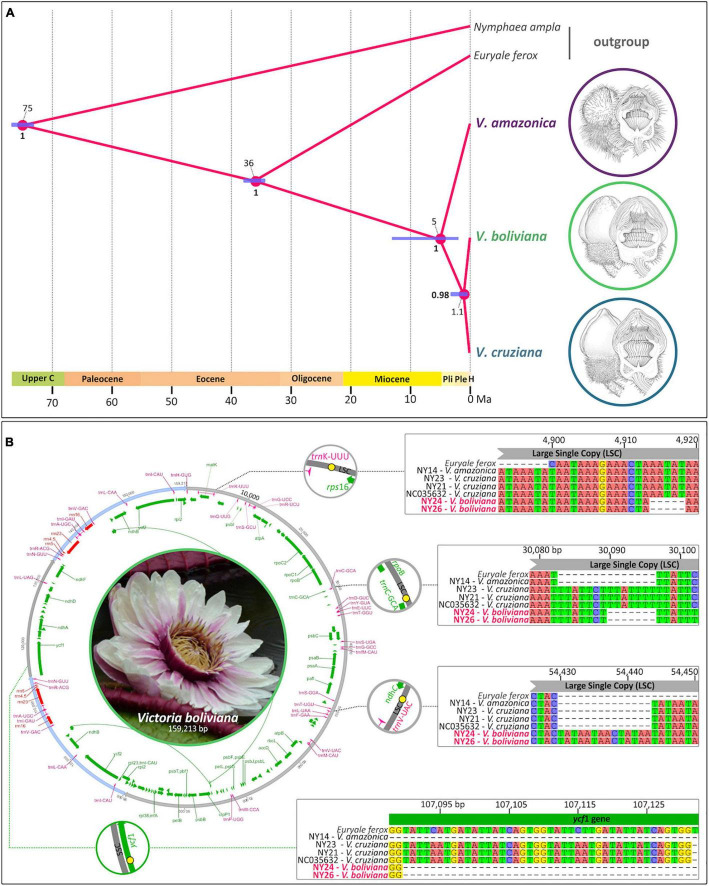
**(A)** Chronogram tree from StarBEAST2 representing molecular dating analysis of splits between *V. amazonica, V. cruziana*, and *V. boliviana* with confidence intervals highlighted. **(B)** Visual representation of plastid structure of *V. boliviana* assembled using GetOrganelle, with genomic locations of diagnostic characters indicated. Details of polymorphisms (indels) – in the context of multi plastid alignments – displayed within insets.

We scored morphological character states from 58 herbarium collection specimens and 175 iNaturalist observations, giving a total of 233 individuals (see Supplementary Tables 4, 5). The congruence of morphological character states to our four morpho species was evaluated by eye, resulting in diagnostic characters being selected (see Table 2 and Figures 4, 5, 7, Key to the species): leaf rim morphology (shape in cross-section, height, color), flower bud apex shape, size and shape of the stigmatic chamber, outer tepal prickle morphology, number and distribution, inner tepal color in bud, carpellary appendage morphology and attachment, seed shape, size and presence / absence of a raphe. These were represented by 25 characters, 13 of which are novel.

### Geographical Observations

We recorded 209 geographical observations assignable to morphospecies (Figure 3 and Supplementary Table 2), 175 of these correspond to iNaturalist records (indicated by * in Supplementary Table 2), and 44 to biological collections in herbaria. In several cases, iNaturalist records extended the distribution of *Victoria* delimited by herbarium collections (Figure 3). For example, in the case of the *cruziana* morphotype, herbarium collections indicate a southernmost limit of the –30.37°S latitude, whilst iNaturalist increased this to –32.92°S. Social media posts provided additional probable distributions for *Victoria* not documented in herbaria or iNaturalist. For example, images of *V. amazonica* from Irinida in Colombia, close to the Irinida and Guaviare rivers, both of which drain into the Orinoco river; and images of *V. cruziana* from the Esteras del Ibera, Argentina.

**FIGURE 8:**
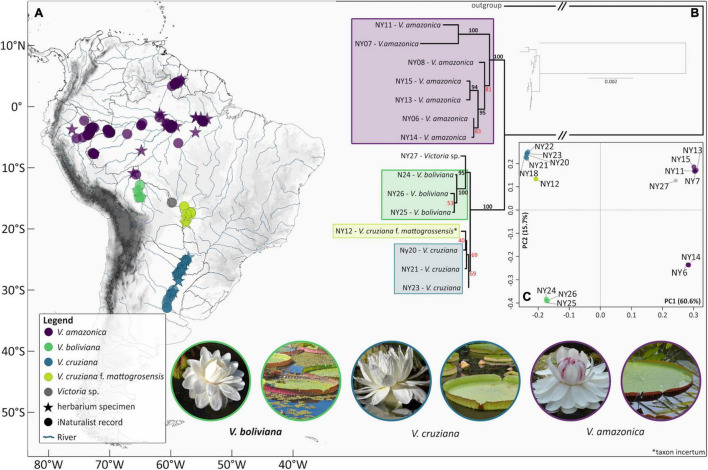
Geographical context with population genomic and phylogenomic results. **(A)** Geographical coordinates of all *Victoria* samples used in analyses, where symbols are color-coded according to morphotype/species and symbol type denotes data source. **(B)** Phylogenomic representation of relationships between plastomes of 15 *Victoria* samples, with *Nymphaea ampla* as the outgroup and constructed using RAxML. **(C)** Principal component analysis of *Victoria* nuclear dataset in 16 individuals based on 229,281 genotype likelihoods obtained after skimming for a minor allele frequency (*maf*) threshold of 0.2.

Our review of herbarium and iNaturalist records (Supplementary Table 2 and Figure 8) and of social media images suggest that *Victoria* is absent from a number of river systems that form part of the Amazon, Essequibo and Paraná river basins (Figure 8 and Supplementary Table 3). The most notable of these being its absence from central eastern and north western Amazonian Brazil and, despite being documented in Colombian tributaries of the Orinoco river, its apparent absence from Venezuela and Ecuador.

### Morphospecies Correlate Well With Geography

Using the geographical observations (Supplementary Table 1) we mapped our four morphospecies across South America (Figure 8). This shows strong congruence between morphospecies and geographical location. Based on our dataset, *amazonica* and ‘*boliviana*’ morphotypes are restricted to mutually exclusive portions of the Amazon river basin, the ‘*mattogrossensis taxon incertum*’ morphotype is restricted to the Pantanal (Paraná river basins), and *cruziana* is restricted to the lower portion of the Paraguay river and Paraná. Despite the presence of both *cruziana* and the ‘*mattogrossensis taxon incertum*’ morphotype on the Paraguay river, occurrence records suggest a large geographical separation between both.

### Distribution

*Victoria* is largely restricted to temperate and tropical Southern Hemisphere South America, not occurring further than 4.2°N and 32.9°S. Whilst it has an extensive temperate distribution in the southern hemisphere, it does not occur in the temperate Northern Hemisphere outside of cultivation.

*V. amazonica* is known from Colombia, Brazil, Guyana, Peru, and Bolivia where it is restricted to the river basins of the Amazon, Guaviare river (a tributary of the Orinoco) and Essequibo rivers. In the Amazon river basin *V. amazonica* occurs in most of the major tributaries except for the Xingu and Madeira rivers. *V. cruziana* is the only temperate species and is restricted to the Paraná River and its tributaries. The *V. ‘boliviana’* morphotype appears to be endemic to the Llanos de Moxos in Bolivia with all the records concentrated in the Mamoré river basin. A photographic record from Rurrenabaque, however, suggests that it may also be present in the neighboring Beni river.

### Genomic Evaluation of Putative Species

#### DNA Sequencing and Mapping of Reads to Plastid and Transcript Datasets

Library insert size, including adaptors, was on average 195 bp for herbarium specimens and 385 bp for fresh collection samples. A total of 3.9-12.5 million raw reads were generated for each sample (Supplementary Table 7). Mapping reads to the plastid genome resulted in an average read depth of 74x (herbarium material) and 1716x (fresh material) (Supplementary Table 8), where the proportion of missing sites was <0.7% for all samples included in the final analyses. As for reads mapped to the set of 37,470 transcripts that constitute our substitute for a nuclear reference genome, their average depth totaled 4x and 29x for herbarium and fresh material respectively (Supplementary Table 9). The length of quality-filtered sequence data aligned to the transcripts totaled up to 36 Mbp in herbarium samples and 43 Mbp in fresh samples. Our BUSCO analysis revealed that our assembled transcriptome captured 1287 (84.7%) of complete BUSCO genes, where 23 (1.5%) were fragmented and 209 (13.8%) missing.

#### Population Genomic and Phylogenomic Analysis of Nuclear Data

For the analysis performed using GLs, the number of positions obtained for each run with different filtering parameters was: 436,329 after *maf* = 0.1 filtering (Supplementary Figure 1) and 229,281 after *maf* = 0.2 filtering (Figure 8). The resulting PCAs suggest that the most informative axis of variation derived from nuclear genomic sequence data of these *Victoria* samples distinguishes *V. amazonica* from *V. cruziana* with *V. boliviana* sp. nov. (40.9 and 60.6% of variation along the 1st PC in respective runs), whereas a smaller proportion of the variation (28.0 and 15.7% in respective runs) collectively describes the segregation of *V. cruziana* from *V. boliviana* sp. nov. as well as the majority of *V. amazonica* from the *V. amazonica* samples from Guyana (NPNY6 and NPNY14). In the *maf* = 0.2 iteration of the analysis, the separation of Guyana *V. amazonica* from the remainder of samples is less pronounced than for the *maf* = 0.1 filtering run. Overall, the spread of samples implies more population genomic structuring within *V. amazonica* compared to that within *V. cruziana.* Regarding the *V. c.* f. *mattogrossensis taxon incertum* sample, based on these nuclear variant sites, it shows an expected affinity of NPNY12 to *cruziana*. The unidentified sample (NPNY27) shows close affinity to the main *V. amazonica* cluster. A very similar pattern of genetic clustering was produced with the *maf* = 0.1 filtering option (Supplementary Figure 1).

#### Phylogenomic Analysis of Plastid Data

Our phylogenomic tree (Figure 8) based on alignments of the entire plastid sequence (with *Nymphaea ampla* and *Euryale ferox* as outgroups) shows strong support for the monophyly of *V. amazonica* samples (100% of BS replicates for this bipartition). Within this clade, there is additionally robust support (94%) for two Brazilian samples (NPNY13 and 15) in the Rio Solimões/Manaus area as well as 100% support for two other Brazilian samples (NPNY7 and 11) provenanced to Municipio de Oriximiná and Ipixuna respectively. The adjacent bipartition (100% support) subtends sister bipartitions including: all of the *cruziana* samples (including *V. c.* f. *mattogrossensis taxon incertum*) (69% support) and all three *V. boliviana* sp. nov. samples along with the unassigned sample NPNY27 (95% support), where the three *V. boliviana* sp. nov. samples have 100% support. Notably, branch lengths are shorter within the *V. cruziana*, *V. c.* f. *mattogrossensis taxon incertum*, *V. boliviana* sp. nov. clade than within the *V. amazonica* clade. The topology derived from the absolute age estimation analysis conducted in StarBEAST2 was in strong agreement with the ML individual level phylogeny. Here, *V. amazonica* was placed as sister to *V. cruziana* and *V. boliviana* sp. nov. with maximum support. The sister relationship of *V. cruziana* and *V. boliviana* sp. nov. was also recovered with strong support (Posterior Probability = 0.98, for HPD intervals, see Figure 7). The analysis further revealed that populations of V. *cruziana* and *V. boliviana* sp. nov. diverged in the Pleistocene, 1.1. Ma, whereas *V. amazonica* branched out from the MRCA of *V. cruziana* and *V. boliviana* sp. nov. 5 Ma (Figure 7).

#### Comparative Genomics of Plastomes Amongst *Victoria* spp.

Four locally collinear blocks (LCBs) were identified by the Mauve alignment. Amongst our set of plastid genomes derived from four species (*E. ferox, V. amazonica*, *V. cruziana*, and *V. boliviana* sp. nov.), no genomic-scale rearrangements were detected, including in the inverted repeat (IR) regions. We detected 12 insertions and deletions differentiating intraspecific *Victoria* genomes, ranging from 4 to 105 bp in size. These included two indels specific to *V. boliviana* sp. nov: a 14 bp insertion in the large single copy region (LSC), two deletions in the LSC (5 and 7 bp in length), the former between *trnK* and *rps16*, and the latter adjacent to *trnC*, and a 42 bp deletion in the coding sequence (CDS) of gene *ycf1*, situated within the small single copy region (SSC) (Table 3).

**TABLE 3:** Plastid polymorphisms (other than point mutations) diagnostic for *V. boliviana* sp. nov. Alignment blocks are arbitrary units computed by Mauve v.2.3.1.

Alignment block	Polymorphism type	*V. boliviana*	*V. cruziana*	*V. amazonica*	Starting position (alignment block)	Plastid region	Proximal genes (flanking unless specified)
LCB1	Deletion	5 bp			4,914	LSC	*trnK rps16*
LCB1	Deletion	7 bp		14 bp	30,090 (30,083 *amazonica*)	LSC	*trnC*
LCB2	Insertion	14 bp			3,499	LSC	*ndhC trnV*
LCB3	Deletion	42 bp		105 bp	17,916 (17,853 *amazonica*)	SSC	*ycf1* (intragenic)
LCB2	4 bp transversion	AAAA	TTTT	TTT-	34,612	LSC	*rpl36 rps11*

*Victoria boliviana sp. nov. deletions and insertion are relative to V. cruziana, where two of the deletions overlap with longer respective deletions in V. amazonica. LSC, large single copy region; SSC, small single copy region.*

Furthermore, a 4 bp transversion unique to *V. boliviana* sp. nov. was found in the LSC. For the three species, seven sample alignment here, DnaSP analysis revealed 182 polymorphic sites, where 17 sites classified as parsimony informative and 8 SNPs constituted alleles private to *V. boliviana* sp. nov. (Supplementary Table 6).

#### Genome Size Estimation and Chromosome Count

The flow cytometric analysis of *V. boliviana* sp. nov. resulted in high resolution flow histograms with the 2C peaks of both the sample and internal calibration standard having low coefficients of variation (CV%) (mean of 2.77 for samples, and 2.99 for calibration standard). Based on the means of the sample G1 and calibration standard G1 peaks, *V. boliviana* sp. nov. has an estimated genome size of 1C = 4.24 pg (Supplementary Figure 2). In addition, a chromosome count of 2*n* = 2*x* = 24 was obtained for the same accession of *V. boliviana* sp. nov (Supplementary Figure 3). The count is identical to that previously determined for *V. cruziana* and different from that for *V. amazonica* of 2*n* = 2*x* = 20.

## Discussion

### Heuristic Delimitation of *Victoria* Species

Through the application of a heuristic multidisciplinary approach, we provide revised species delimitations and diagnoses for *Victoria*. This has resulted in the recognition of a species new to science: *V. boliviana* Magdalena and L. T. Smith and highlighted the morphological distinctness of *V. cruziana* forma *mattogrossensis taxon incertum*. Species delimitation and nomenclatural stability in *Victoria* have until now been hampered by the loss of the original type material that served to fix the species name as well as a paucity of biological collections. This resulted in disagreement over the number of species recognized (Pellegrini, 2020; de Lima et al., 2021), the application of an incorrect name for *Victoria amazonica* for most of the 19th and 20th centuries (Prance, 1974) and a failure to recognize taxa in this iconic genus. Nevertheless, the recent selection of a neotype for *V. amazonica* and a lectotype for *V. cruziana* (de Lima et al., 2021) has helped to underpin nomenclatural stability and anchor species delimitation with respect to morphology. Here, we sought to delimit *Victoria* species, through the development of a heuristic and iterative approach – one which integrates field, horticultural, morphological and genomic observations and analyses. In the primary iteration of this investigation, we used geographical observations to integrate morphological observations from biological (herbarium) collections with those from field observations (iNaturalist, horticultural observations). In doing so, we were able to recognize discrete morphological units within a heuristic species concept that focuses on cohesion rather than divergence. These formed *prima facie* null hypotheses that we tested using genomic observations. Based on this approach, we recognize three discrete units at the rank of species, and provide strong justification for further research into a fourth taxon of unknown rank. Our morphological species delimitation differs from that of de Lima et al. (2021) who propose considering all populations of *Victoria* as a single species, *V. amazonica*. Their conclusion was based on the interpretation of the resulting large variation in the morphological characters observed in their single species as being the product of its broad spatial distribution and aquatic habit.

### Overcoming the Challenge of Small Numbers and Poorly Preserved Biological Collections

Past studies (e.g., de Lima et al., 2021) have been limited by the small number of herbarium collections available to study and their state of preservation, both of which are likely a product of the large size and fleshy nature of the plants. We overcame this through the use of high-resolution specimen scans available online, supplemented by “research quality” geo-referenced iNaturalist field images and observations of horticultural material. Using iNaturalist and digitized herbarium specimens we were able to incorporate 233 collections of *Victoria*, a significant increase on previous studies based on herbarium specimens alone (de Lima et al., 2021). iNaturalist images and observations allowed for characters usually lost in herbarium such as leaf rim morphology, the color of leaf blades, flower bud apex shape, the distribution and shape of prickles on the outer tepals, and the color and shape of tepals (Table 1).

The geo-location of *Victoria* lily pads enabled us to use these records to undertake assessments of extinction threat and so greatly increase the accuracy of those assessments. Whilst we did not use social media accounts as a source of georeferenced localities, we did use them to identify potential gaps in our knowledge of *Victoria*’s distribution. Instagram, which is image-based, was particularly useful in indicating the presence of *Victoria cruziana* in the Esteras del Ibera wetlands (Argentina), suggesting that it occupies a broader swathe of south eastern South America. Instagram also suggested the presence of *V. amazonica* in the Orinoco, and the cultivation of *V. cruziana* f. *mattogrossensis taxon incertum* under the names *V. amazonica* or *V. regia* in Brazil.

### Diagnostic Morphological Characters for Delimiting *Victoria* Species

We identified the morphology of the leaf rim, flower prickles, the stigmatic chamber, carpellary appendage shape and size, and the seeds, as phylogenetically informative diagnostic characters in *Victoria*. Of these, we are the first to propose stigmatic chamber, carpellary appendage and seed morphology. This was surprising as these characters are all readily observable and prominent features that would be obvious to an experienced observer. A possible explanation may be that there was confusion over species delimitation stemming from d’Orbigny’s (1840) mistaken diagnosis of *V. cruziana* against *V. boliviana* sp. nov., and not *V. amazonica*, as he and subsequent authors supposed. In the absence of type material or an independent class of observations this would have been difficult to resolve.

The prickles on the abaxial surface of the outer tepals have not been proposed as diagnostic in *Victoria* since Malme (1907). Based on our observations, *Victoria amazonica* always has prickles which taper smoothly to a point at their apex (Figure 4) and are distributed relatively evenly over the entire tepal surface. By contrast, in *V. cruziana* (Figure 6) the prickles are absent or relatively sparsely distributed, but, where present, they taper abruptly to a point and are distributed over only the basal third of the tepal, whilst in *V. boliviana* sp. nov. (Figure 5) prickles are usually absent but, if present, may be found anywhere on the tepal surface and taper abruptly to a point. We speculate that prickles play a defensive role and protect the relatively nutrient rich contents of the bud and the protein-rich beetles trapped within at anthesis but are unable to account for the variation between species in the genus given the incomplete and small number of studies focusing on *Victoria* biology or autecology.

We also discovered that the size and shape of the stigmatic chamber, and of the carpellary appendages which surround and heat it, differs between species (Table 2). As above this suggests a link to pollination but again, given that the pollination-biology of *Victoria cruziana* and *V. boliviana* sp. nov. is very poorly known, it is only possible to speculate that the size and shape of the stigmatic chamber may be responding to differences in pollinator type, size or number, whilst differences in the size and disposition of the carpellary appendages may be products of the need to accommodate a different size of stigmatic chamber, or to produce greater or lesser amounts of heat in relation to ambient temperatures or pollinator preferences.

We found observable differences between the seeds of all three species with respect to their shape and size, and of the prominence of the raphe. *Victoria amazonica* has ellipsoid seeds compared to the globose seeds of *V. cruziana* and *V. boliviana* sp. nov. *V. boliviana* sp. nov. has relatively large seeds with a prominent raphe, compared to *V. cruziana* and *V. amazonica*. The significance of seed shape is unclear but may be related to dispersal through the gut of an unknown disperser, an ellipsoid seed being easier to pass than a globose one. Whilst no evidence of endochory has been found, given the lack of research it should not be excluded. Seed size has also been associated with the establishment depth of *Nymphaea* (Jacobs and Hellquist, 2011) suggesting that smaller-seeded species establish in shallower water. This would concur with the observations of previous authors (Prance and Arias, 1975; Rosa-Osman et al., 2011, Magdalena, personal observation) and suggest that *V. boliviana* sp. nov. establishes itself at greater water depths than *V. amazonica* and *V. cruziana*. Finally, similarly to *V. cruziana*, *V. boliviana* sp. nov. develops more rapidly than *V. amazonica* (Magdalena, personal observation.). As above, these aspects remain to be further studied.

### Cohesion and Distinctness Characterizing Genomic Datasets Support *Victoria* Species Hypothesis

We explored complementary concepts of genomic distinctness (in the form of genetic clusters) and divergence of evolutionary lineages in order to test whether the identified morphotypes were corroborated by molecular evidence. At this shallow taxonomic scale, a PCA has more power to highlight distinctness in nuclear genomic data than phylogenomic methods; the latter can be confounded by ILS (Lissambou et al., 2019). Even with our genome skimming approach, given the size of the *Victoria* nuclear genome [1C of 4.66 (*V. amazonica*), 4.10 (*V. cruziana*) and 4.24 (*V. boliviana* sp. nov.)], which is at least double the size of most Nymphaeales species profiled using flow cytometry (Pellicer et al., 2013), the capacity to retrieve appropriate nuclear genes at high coverage for phylogenomic inference was limited. Our response was to retrieve genotypes by mapping to curated transcriptomic data and to summarize this genetic variation using a dimensionality-reduction method. The tight respective clusters of *V. cruziana* and *V. boliviana* sp. nov. on the PCA and their degree of separation along the 2nd axis of variation serves to demonstrate their genetic distinctness. Importantly, this distinctness could be due to geographical isolation alone, which is why such results must always be assessed against other lines of evidence, such as morphological differences. Our PCA additionally demonstrates a greater degree of genetic structuring and variation within *V. amazonica* compared to *V. boliviana* sp. nov. or *V. cruziana*. This is concordant with the broader geographical spread of *V. amazonica* in northern South America, though we also note the larger sample size of *V. amazonica* available for this analysis. Furthermore, we would expect to see more continuity of the *V. amazonica* cluster on the 2nd PC, linking the two samples from Guyana (NPNY6 and NPNY14) and the samples from Brazil, had we been able to genotype a more broadly geographically sampled set of accessions. Implementation of a population genetic framework using high throughput sequencing (HTS) datasets to support molecular-based species delimitation is not a widespread practice, but is gaining some traction in studies of plants. For example, where they occur in sympatry (Ikabanga et al., 2017) or where taxonomic incongruity is prevalent within the genus (Rodríguez-Rodríguez et al., 2018; Rutherford et al., 2018). Our study demonstrates a novel way to apply this approach – in the absence of a reference genome, but utilizing an available transcriptome.

The dataset of mapped full plastid genomes suggests a similar conclusion in terms of delimiting separate evolutionary units of *Victoria*. This is presented as a strongly supported monophyly of respective clades containing species *amazonica*, *boliviana* sp. nov. and *cruziana*. The longer branch lengths of samples within the *amazonica* clade also suggests ancient differentiation. Our molecular dating suggests that plastid populations in Victoria diverged ∼5 Ma, with the time of divergence of *V. boliviana* sp. nov. and *V. cruziana* set to have occurred as recently as 1.1 Ma.

An unresolved question is the degree to which hybridisation and introgression might have been involved in the evolution of *V. boliviana* sp. nov. By revealing that this species has a chromosome count identical to that of *V. cruziana*, a hybrid origin cannot be easily supported. Even though the genome size of *V. boliviana* sp. nov is intermediate between that of *V. cruziana* and *V. amazonica*, additional molecular processes such as repeat amplification and chromosome rearrangements have almost certainly been involved its genome evolution, especially given its divergence time from *V. cruziana*.

### The Plastid as a Source of Molecular Characters for Species Diagnosis

Finally, we supplement these lines of evidence derived from different cellular compartments with genomic features identified in the assembled chloroplasts that we propose to be diagnostic to the new species. The absence of large-scale genomic rearrangements in our plastid genomes was not surprising given that gene order in the chloroplasts of Nymphaeales has been found to be conserved (Gruenstaeudl et al., 2017). The three indels unique to *V. boliviana* sp. nov., could be used to support the molecular identification of *Victoria* specimens. The longest indel was found in the *ycf1* gene (105 bp in *V. amazonica* and 42 bp in *V. boliviana* sp. nov.). *Ycf1* is a large housekeeping gene (Drescher et al., 2000) involved in photosystem biogenesis (Yang et al., 2016). Due to its high variability (Dong et al., 2015), *ycf1* has been highly utilized in phylogenetics (Neubig et al., 2009; Dastpak et al., 2018). Here, we shed light on the utility of this gene as a potential tool for DNA barcoding at shallow phylogenetic scales. One application could be in genome skimming studies, where retrieval of full chloroplast genomes is routine. A low-cost alternative is a simple PCR of this genomic region, where, due to length differences, gel electrophoresis could be used for species identification. Diagnoses of new taxa that incorporate DNA-based characters are not common, but can be more useful than lineage-based diagnoses, especially when applied to known or type specimens. They are also an unbiased means of species delimitation (Renner, 2016). A small number of previous studies have used diagnostic molecular characters e.g., nucleotides at certain positions of *matK* and *trnL-trnF* regions in *Buxus* spp. (Buxaceae; Gutiérrez et al., 2011) and of *nhdF* and the *ITS* region in *Brunfelsia* (Solanaceae; Filipowicz and Renner, 2012). Length variation associated with indels has been previously applied as a form of diagnostic genetic variation – for example to the study of *Abies* (Xiang et al., 2018). Our approach has extended this to examination of the whole plastome.

### Suggestions for Future Research Priorities in *Victoria*

Ensuring that species of *Victoria* remain for future generations requires that risks of extinction can be accurately evaluated and monitored. In the case of *Victoria* this requires greater knowledge of the species’ natural history and autecology, specifically their pollination and dispersal biology – against which potential threats can be evaluated – and the extent and fluctuation of population sizes.

This is important as it enables threats to the viability of populations to be evaluated. Knowledge of both is at best superficial and based on a small number of field observations of *V. amazonica* and *V. cruziana* (Schomburgk, 1837; Archangeli, 1908; Prance and Arias, 1975; Hanagarth, 1993). The dispersal biology of *Victoria* is poorly known and based largely on speculation rather than observation of tested hypotheses. There is also no literature on the size, fluctuation and connectivity of populations of the three species. A viable approach for doing so would be to use publicly available time-stamped remote-sensed data, such as Google Earth image layers to monitor populations given that the lily pads can be seen in higher resolution images.

Our small molecular sample set recovered genetic structure within *V. amazonica* (Figure 8) and we would argue for greater sampling of the genus, especially for the edges of its Amazonian range (Colombia, Guyana, Peru, Venezuela) and in the vicinity of the Pantanal. Additionally, since morphological observations suggest a fourth species (*V. c.* f. *mattogrossensis taxon incertum*), strategic sampling for genomic work may result in the molecular support for it at the rank of species.

Establishing the status of *V. c.* f. *mattogrossensis taxon incertum* should be seen as a high priority. Should it be evaluated as a distinct species, it would be one of the most vulnerable to extinction, having the smallest range and occupying a region that has been impacted by extreme drought during the last decade (Marengo et al., 2021).

Finally, a larger sample set would allow for investigation of the barriers to dispersal within the genus and extent of gene flow between the different populations of *Victoria* species.

Understanding the latter would also require the generation of more extensive nuclear molecular datasets and a more contiguous genome of reference. Construction of a nuclear phylogenomic framework would enable the computation of introgression tests based on patterns of allele sharing between taxa (e.g., D statistics; Durand et al., 2011) and permit a more nuanced investigation of the evolutionary history of these aquatic plant species, including investigation of forces driving speciation of *V. boliviana* sp. nov.

The above research would require improved sampling of the species’ distributions, underpinned by verifiable biological (herbarium) collections. Combined with the need for increased natural history, ecological and genetic observations we would propose that the genus be the focus of a dedicated field campaign.

*Victoria* also has great potential to serve as a valuable model for exploring the biogeography of continental South America as it is an aquatic species mostly restricted to large river systems; its seeds are desiccation intolerant and thus unable to escape flooding plains of their water catchment. The ephemeral nature of such flooding plains (Cowgill and Prance, 1989) is thought to have driven gigantism, as a mode of outcompeting other aquatic plants (Box et al., 2022). *Victoria* could feasibly be part of a monophyletic clade comprising *Microvictoria*-*Euryale*-*Victoria*, as is suggested by morphological observations (Gandolfo et al., 2004), the age of which predates the complete break up of Gondwana ca 83 Ma (Seton et al., 2012). The current distribution of genus *Victoria* spans a vast area of river systems, representing ca 44% of the South American drainage basins (ca 7.8 × 10^6^ km^2^) in a region where orogenesis and changing climates have wrought major changes in the last 12 Ma (Antonelli et al., 2009; Figueiredo et al., 2009; Hoorn et al., 2010, 2022).

## Taxonomy

### Key to the Species

1. Mature leaves with upturned rim, rim moderate to high (8–10% of blade length), sigmoid in cross- section; mature bud concave towards apex; carpellary appendages with a cuneate base, arising 45° from the point of attachment (see Figure 2); prickles associated with the flowers tapering abruptly to a point, covering ovary and either absent, or covering the basal 1/3 of abaxial surface of the outer tepal, prickles 0 – 100 per tepal; seeds globose, raphe faintly visible. Paraná river basin, lower course of Paraguay river *V. cruziana* (Figure 6)
1. Mature leaves with no upturned rim, or where present the upturned rim low to moderate (4–7% of blade length), sigmoid or vertical in cross-section; mature bud convex towards apex; carpellary appendages with an auriculate or subauriculate base, arising 45° from point of attachment or not (see Figure 3); prickles associated with the flowers tapering smoothly or abruptly to a point, covering both the ovary and entire abaxial surface of the outer tepal, or only the ovary, 0–1000 prickles per tepal; seeds ellipsoid or globose, raphe faintly visible or prominent. Amazon river basin, Pantanal 2
2. Mature leaves with no or moderate upturned rim, which, where present, is vertical in cross-section. Prickles associated with the flowers tapering smoothly to a point and covering both ovary and entire abaxial surface of the outer tepals, 55–300 prickles per tepal; stigmatic chamber deeply concave, obdeltate in longitudinal profile, carpellary appendage auriculate and hanging free from, not arising 45° from point of attachment (see Figures 2, 3); seeds ellipsoid, the raphe faintly visible. Amazon river basin excluding *V. amazonica* (Figure 4)
2. Mature leaves with a moderate upturned rim, which is sigmoid in cross-section. Prickles associated with the flowers tapering abruptly to a point, covering both the ovary and entire abaxial surface of the outer tepals, or only the ovary, 0–1000 prickles per tepal; stigmatic chamber shallowly concave and oblong in longitudinal profile, carpellary appendage subauriculate at point of attachment not arising 45° from point of attachment (see Figure 3) (not known for *V. cruziana* f. *mattogrossensis*); seed ovoid, the raphe prominent. Llanos de Moxos or Pantanal 3
3. Prickles associated with the flowers covering the ovary and either absent from or sparsely distributed over the abaxial surface of the outer tepals, 0–10 per tepal; apical portion of the carpellary appendage longer than the basal portion. Llanos de Moxos *V. boliviana* (Figure 5)
3. Prickles associated with the flowers covering the ovary and distributed densely and evenly over the abaxial surface of the outer tepals, 500–1000+ per tepal; apical portion of the carpellary appendage shorter than the basal portion. Pantanal *V. cruziana* f. *mattogrossensis taxon incertum taxon incertae*

**Victoria** R. H. Schomb., *Athenaeum* (London) 1837 (No. 515): 661 (September 9 1837).

*Victoria* Lindl., Monog. 3 (October 16 1837).

*Victoria* J. E. Gray, Mag. Zool. Bot. 2(10): 373 (December 1 1837).

Aquatic perennial herb, rhizome erect, tuberous, elongate to cylindrical, roots adventitious. Leaves floating, orbicular, peltate, perforated by stomatodes, adaxial surface of lamina glabrous, lacking prickles, green; abaxial surface of lamina with prominent radial and reticulate ribs, juvenile leaves sagittate; leaf margins flat or upturned; prickles covering petiole and ribs. Inflorescences uniflorate, bracteate. Flowers axillary, solitary, multiple buds per plant; pedicel with 4 primary air chambers, 8 minor chambers, covered in prickles; flowers opening one at a time, projecting above water surface shortly before anthesis, projecting above or resting on water surface at anthesis, each flower opening over two nights and partially closing in between, protogynous. Epigynous, ovary globose, covered in prickles externally, ovules parietal, attached by short funiculi, globose. Outer tepals 4, triangular, apex acute to rounded. Inner tepals 40–c.100, arranged in spiral series, creating a torus (attachment point of tepals forming a ring of tissue), tepals gradually reducing in size towards the center and changing shape from apically rounded to acute from outer to innermost; outer staminodia in 1 or 2 whorls, thick, rigid, apiculate; stamens > 100, borne in c. 3 series, subulate, introrse; anthers linear-elongate; inner staminodia, >50, sigmoid, subulate, partially adnate to carpellary appendages, detaching at second-night anthesis; carpellary appendages L-shaped, arising from extension of stigmatic surface, lower parts adnate to tissue extending from tepal base attachment, corresponding in position and number with stigmatic surface ridges and locules. Fruit ripening just below surface of water, 10–15 cm in diameter (excluding prickles) at maturity, fleshy, oblate, topped by a shallow cylinder-shaped mass of dark reddish to maroon ring of persistent hard tissue formed by the remnant bases of tepals; inner staminodia persistent and curved over concave stigmatic surface while ripening; outer layers of pericarp disintegrating to release seeds. Seeds smooth, surrounded by a mucilaginous aril.

Three, possibly four species, tropical and temperate South America.

**FIGURE 9:**
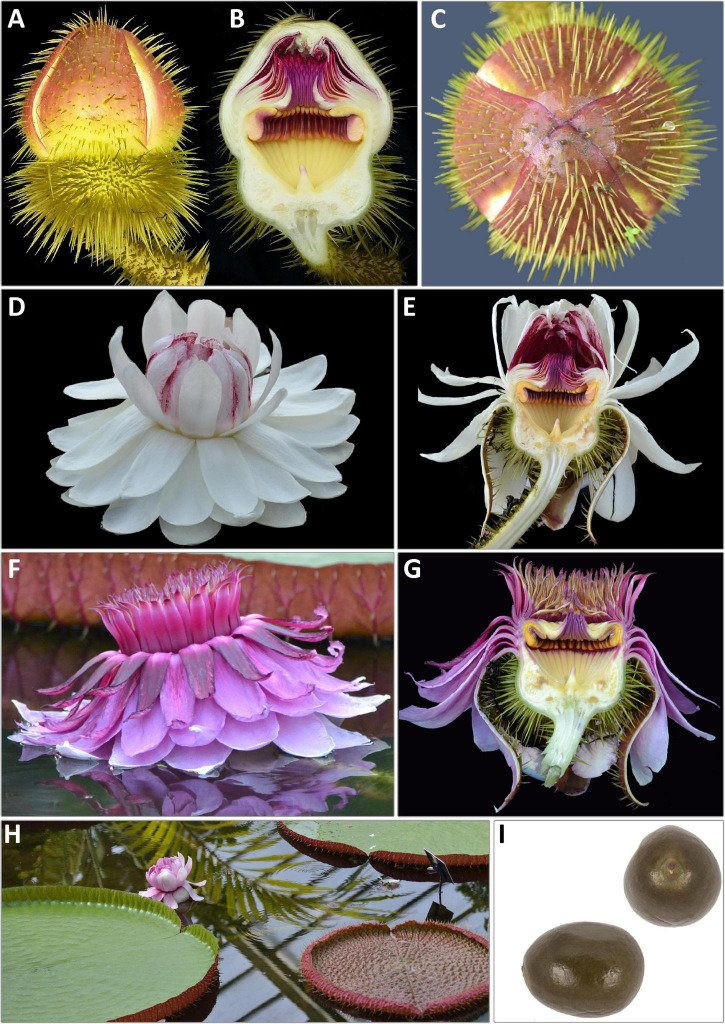
*Victoria amazonica* **(A)** bud whole, **(B)** bud L.S., **(C)** bud from above, **(D)** first night flower, **(E)** first night flower L.S., **(F)** second night flower, **(G)** second night flower LTS, **(H)** habit, and **(I)** seed. **(A–H)** (LTS) and **(I)** (CM) cultivated RBG Kew.

**Victoria amazonica** (Poepp.) Klotzsch, *Bot. Zeitung (Berlin)* 5: 245 (1847). *Euryale amazonica* Poepp. *Froriep’s Not. Natur- Heilk*. 35: 131 (1832). Type: *Poeppig s.n.* (holotype W -presumed destroyed in WWII); Brazil, Amazonas, Careiro da Várzea [Teresina], Ilha de Careiro, 25 Sept. 1974, *G.T. Prance* 22745 (neotype (selected by de Lima et al., 2021): INPA (INPA46745); isoneotypes: K (K000837777!), NY (NY2269910, NY2269911, NY2269928), MO (MO3414212), US (US01341606)). Vernacular names: Forno de Jaçanã, Auapé yapóna, *Victoria regia*, Giant Amazonian Waterlily. Figures 1A, 2, 3A–C, 4, 9.

*Victoria regina* R.H.Schomb. Athenaeum (London) 515: 661 (September 9, 1837).

*Victoria regia* Lindl., Monograph: 3 (October 16, 1837). Type: Victoria Regia: 3, Plate 1 (October 16, 1837). *nom. superfl.*

*Victoria regina* J. E. Gray, *Mag. Zool. and Bot*. 2(11): 440 (December 1, 1837). *nom. superfl. Victoria reginae Hook. Hooker’s J. Bot. Kew Gard. Misc*. 2: 314 (1850). *orth. var.*

*Leaves* up to 2.3 m broad, adaxial surface of lamina green, occasionally tinged bronze in younger leaves; abaxial surface of lamina maroon or green, radial and reticulate ribs maroon, yellow or green; leaf margins form a low to moderate rim c. 4–7% of the lamina length (higher in crowded habitats), rim curved at its base then ± perpendicular to adaxial surface, abaxial surface of rim maroon or green; hairs 0.3–12 mm, simple, multicellular, 3–12 segmented. *Flowers* up to 28 cm in diameter at second-night anthesis. Ovary 8–12 cm diameter, outer surface covered in prickles 1– 18 mm (dried), prickles gradually tapering to a sharp point, hairs absent or present, where present simple, 0.1–0.4 mm, inner surface of ovary with deeply concave stigmatic surface, rounded to triangular in longitudinal profile, ridged with lines corresponding with 25–36 radially arranged locules, each containing 25–28 ovules, 1–1.5 mm diameter (fresh). Outer tepals 4.9–12 × 4–8 cm when fresh; abaxial surface predominantly brown/maroon, bearing 55–330 prickles per tepal, prickles tapering gradually to a sharp point, ranging from 1–14 mm (dried), spaced regularly, irregularly, or clustering more densely toward the base over entire surface, hairs absent or present on abaxial surface, where present 0.1–0.2 mm. Inner tepals 7–15 × 2–6 cm (fresh), innermost deep maroon in bud; all others remaining white or turning pink to dark pink at second-night anthesis; outer staminodia > 25, 5–6 × 1–1.5 cm thick, rigid, apiculate; stamens 2–4 × 0.5–1 cm; inner staminodia, 4–6 × 0.5–1 cm; base of lower parts of carpellary appendage auriculate/rounded in shape and hanging free from extension of stigmatic surface, length of upper parts not exceeding that of lower parts. *Flower at first night of anthesis*, inner tepals white, with innermost tepals dark maroon, outer staminodia tipped pink; *second night anthesis*, innermost tepals dark maroon, inner tepals remaining white or pink to dark pink or red, darkest at base, outer staminodia remaining white or dark pink for basal two thirds of their length, tipped pink, inner staminodia pink at base. *Seeds* 600–1000 per fruit, 7–8 × 9–10 mm, ellipsoid, green to brown, raphe faintly visible.

**Distribution and Conservation Status —***Victoria amazonica* is restricted to the Amazon river basin, from Northern Brazil, Bolivia, Colombia, Guyana and Peru. Its EOO is estimated to be 2,640,795 km^2^, exceeding the threshold for an IUCN threat category under criterion B, whilst its AOO is estimated as 476 km^2^, falling into the Endangered category. We believe that our calculation of the AOO is likely an underestimate, resulting from difficulties in observing the species in the field and under-representation of the genus in biological collections.

There are more than 10 locations for which threats have been assessed, but there is not the information on population fragmentation or fluctuation available in order to be able to assess the ‘severely fragmented’ and ‘extreme fluctuations’ subcriteria. A continuing decline in habitat quality is inferred due to the presence of hydroelectric dams, mining, and deforestation of the river systems from where *V. amazonica* is documented. For example, in Peru, localities along the Marañón river to the headwaters of the Amazon and the Ucayali river have been heavily deforested by gold mining (i-Terra, 2021). Gold mining is associated with profound mercury contamination of rivers and aquatic species (USGS Environmental Health Program, 2019) and increases in Peruvian mercury imports suggest that contamination must be increasing (Swenson et al., 2011). In Brazil, as in Bolivia there have also been reports of mining activities in indigenous lands adjacent to several *Victoria* populations (Hutukara Associação Yanomami and Associação Wanasseduume Ye’kwana, 2020; INPE, 2021; MapBiomas, 2021; Mercado, 2021).

*Victoria amazonica* is here assessed as Least Concern (LC) considering that its range stretches across Amazonia and currently exceeds the parameters for a threatened category under criterion B. We note, however, a moderate number of locations where populations are under threat and there is a continuing decline in habitat quality. Further investigation and surveys are needed to better understand trends in population size, fragmentation, distribution and the impact of climate change.

**Notes.** *Victoria amazonica* is the only species whose leaves do not always form an upturned rim, and when they do, it is usually low and vertical in profile rather than recurving over the flat part of the lamina. Its flowers are distinguished from *V. cruziana* and *V. boliviana* both in bud and on first-night opening, as the innermost tepals are dark maroon rather than white. Carpellary appendages are curved at the base of the lower part and hang freely away from the attachment point. The prickles covering both outer tepal abaxial surface and outer ovary are uniquely gradually tapering to a sharp point (not abruptly tapering as in other species), and always cover the entire abaxial surface of the outer tepals. Its seeds are ellipsoid rather than globose.

**Material Examined**— BOLIVIA. **Pando:** Manuripi: pond north of Rio Madre de Dios., –66.126667, –10.903611, 09/07/1997, *Ritter, N., Crow, G.* & *Crow, C. 4170* (LPB, MO). BRAZIL. ‘North Brasil’:

1898, *Vaughan, G. 61* (K). **Acre:** Río Moa, margem esquerda; lugar chamado Humaita, –72.895556, – 7.614444, 01/10/1984, *Ferreira, C. A. 5123* (NY). **Amapá**: Itaituba, Igarape no Rio Tapajos, – 55.961111, –4.229167, 16/12/2017, *Brogim, R. 4* (UPCB). **Amazonas:** Ipixuna, Margem do Rio Croa, –72.556667, –7.745278, 15/02/2009, *Quinet, A., Saraiva, B., Firmeza, T.1582* (K, SPF); Teresina, Ilha de Careiro, –59.81667, –3.1, 25/09/1974, *Prance, G. T. 22745* (K, NY, US); Basin of Rio Purus area. Lago Preto, 3 km north of Labrea, –64.813056, –7.229772, 29/10/1968, *Prance, G. T., Ramos, J. F. and Farias, L. G. 8016* (NY); Rio Solimões, south bank near Carreiro, –59.806667, –3.168889, 05/02/1974, *Steward, W. C. And Ramos, J. F. P20211* (K, NY, US); Rio Amazonas, from Manaus to 100 km lower reaches, –59.141944, –3.216111, 08/08/1987, *Tsugaru, S. and Yotaro Sano B-769* (MO, NY); Ilha do Cantagalo, –61.503889, –1.570833, 04/07/1995, *Adalardo-Oliveira, A. 2645* (NY, SPF); Riverside and small islets of Rio Solimões within 100 km upper-stream from Manaus, –60.728056, 3.260278, 15/08/1987, *Tsugaru, S. and Yotaro Sano B-1069* (NY); **Pará**: Santarem, Igarape, Ilha Grande de Santarum, –54.706840, –2.450070, –/10/1849 and –/11/1849, *Spruce, R. 441* and *Spruce s.n., s.d.*, (K, M, P), – /04/1850(NY) 1849 (P); Oriximina, Lago Uraria, SW of Orixima, across Rio Trombetas, – 55.9025, –1.812778, 11/06/1980, *Davidson, C. and Martinelli, G. 10241* (MO, NY, RB, US); Rio Cupari, Lago Curuca, 01/01/1948, *Black, G. A. 48-2223* (IAN); Rio Cupari, Lago Curuca, 02/01/1948, *Black, G. A. 48-2253* (IAN); Rio Cupari, Lago de Curuca, 02/01/1948, *Black, G. A. 48-2254* (IAN, NY, US); Oriximina, Rio Trombetas, Lago Ururia, 6 km SW de Oriximina, –55.910278, –1.803889, 08/06/1980, *Martinelli, G. 6945* (RB); Pacoval, Rio Curua, –55.083333, –1.833333, 6–8/08/1981, *Jangoux, J., and Riberio, B. G. S. 1647* (NY); Santarem, 25/12/1938, *Markgraf 3873* (RB); Monte Alegre, Rio Gubatuba, proximo a vila Pare Sol, –54.043333, –2.011944, 17/07/2011, *Lima, C. T. 503* (HUEFS); Pacoval, Rio Curua, –55.083333, –1.833333, 6–8/08/1981. **Roraima:** Rorainopolis, Rio Branco, Lago do Pirarucu, 25 km antes da boca com o Rio Negro, –61.8525, –1.158333, 28/03/2012, *Martinelli, G., Moraes, M. A., Benevides, P., Forzza, R. C., Nadruz, M., Gallucci, S., Costa, D. 17700* (RB). COLOMBIA. **Amazonas**:Leticia, below Quebrada de Arara, –70.065278, –4.05944, 28/01 – 07/02/1969, *Plowman, T., Lockwood, T., Kennedy, H., Schultes, R. E. 2313* (K). GUYANA. **Berbice:** Berbice, –58.2778, 4.394033, 1837, *Schomburgk s.n.* (K). **Upper Takutu-Upper Essequibo**: Karanambo, Rupununi River, –59.3, –3.75, 27/09/1988, *Maas, P. J. M., Koek-N, J., Lall, H., ter Welle, B. J. H., Westra, L. Y. 7727* (K). PERU. **Maynas:** East of Puerto Alegria, –70.0625, –4.103611, 15/03/1977, *Gentry, A. and Daly, D. 18351* (MO); Isla Padre (Cocha Paster), –76.166667, –3.75, 21/12/1982, *Vasquez, R.., Grandez, C. and N. Jaramillo, N. 3684* (MO); Padre Isla in Río Amazonas, and in the cato below Iquitos., –73.163611, –3.651944, 22/05/1978, *Gentry, A., Jarmillo N. 22133* (MO). See Supplementary Data.

**FIGURE 10:**
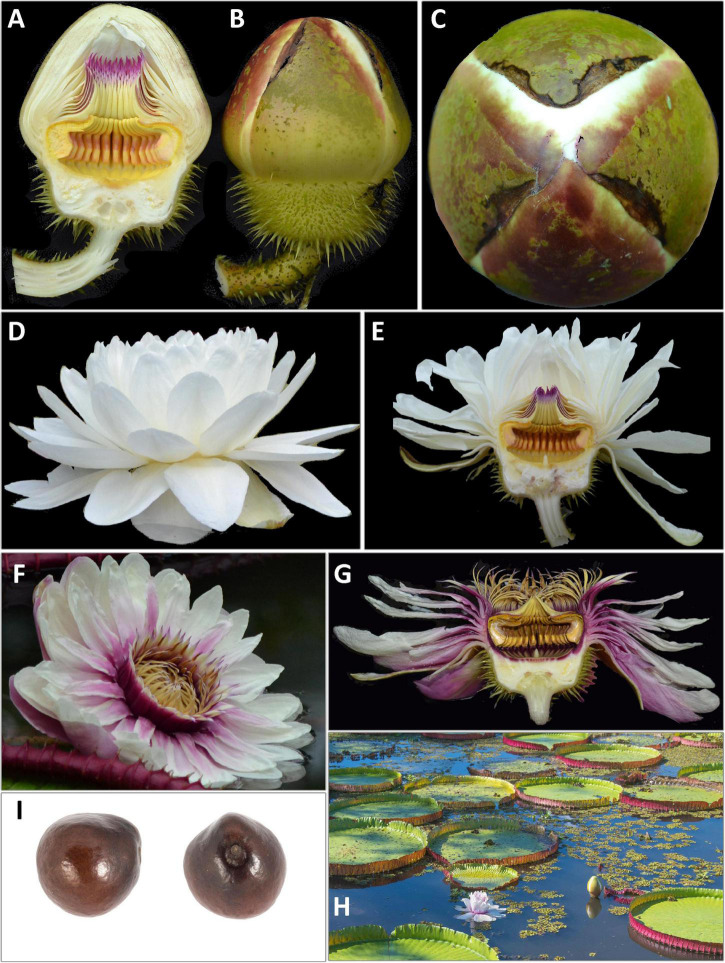
*Victoria boliviana* sp. nov. **(A)** bud whole, **(B)** bud L. S., **(C)** bud from above, **(D)** first night flower, **(E)** first night flower L. S., **(F)** second night flower, **(G)** second night flower L.S., **(H)** habit, and **(I)** seed. **(A–G)** (LTS), **(I)** (CM) cultivated RBG Kew, H (CM) Beni, Bolivia.

**Victoria boliviana** Magdalena and L. T. Sm., ***sp. nov***. Type: Bolivia, Beni Department, Provincia Ballivían, subiendo el Río Yacuma desde Puerto Espíritu, laguna en conexíon al Río Yacuma, unos 20 m al N, 29 Mar. 1988, *S. G. Beck 15173* (holotype: LPB; isotype: K (K000798309). Vernacular names: Reina Victoria, Victoria regia. Figures 1B, 3D–F, 5, 10.

Most similar to *V. cruziana* Orb., from which it can be distinguished by the lower rim of the floating leaf, convex apex of the flower bud, length of the upper part of the carpellary appendages exceeding that of the lower part and the larger seeds. The *V. boliviana* plastid genome differs from that of other *Victoria* species by a 14 bp insertion between plastid genes *ndhC* and *trnV* in the large single copy region (LSC), a 5 bp deletion between *trnK* and *rps16*, a 7 bp deletion adjacent to *trnC* in the LSC and a 42b p deletion in the CDS of gene *ycf1*, within the SSC. Finally, a 4 bp transversion unique to *V. boliviana* sp. nov. was found in the LSC.

*Leaves* up to 3.2 m broad, adaxial surface of lamina green; abaxial surface of lamina dark green, maroon or dark-blue, radial and reticulate ribs yellow or green; leaf margins upturned to form a moderate rim c. 4–7% of leaf length, rim recurving strongly over blade surface at base and curving inwards or flared outwards at top, abaxial surface of rim deep maroon or very pale green/white in color, glabrous or with hairs, where present 1.2–3 mm, simple, multicellular, 6–15 segmented. *Flower* bud broadly ovoid, convex at apex, up to 36 cm in diameter at second-night anthesis. Ovary 8–10 cm diameter, outer surface covered in prickles, 1–10 mm (dried) glabrous; prickles abruptly tapering from c. half of length to sharp apex; inner surface of ovary with shallowly concave stigmatic surface, oblong in longitudinal profile, ridged with lines corresponding with 25–36 radially arranged locules, each containing 8–14 ovules, 2–2.5 mm diameter (fresh). Outer tepals 4, 10–15 × 8–10 cm when fresh, abaxial surface predominantly green, or tinged maroon, prickles absent or present, where present up to 10 per tepal, prickles tapering abruptly at their midpoint to a sharp point, 1–10 (dried), distributed irregularly over entire surface, glabrous. Inner tepals 6–15 × 1.5–9 mm (fresh), innermost remaining white or turning pale pink at their base at the second-night anthesis; outer staminodia > 50,3–4 × 0.5– cm, thick, rigid, apiculate; stamens, 4–5 × 0.5–1 cm; inner staminodia 4– 5 × 0.5–0.7 base of lower parts of carpellary appendage angular in shape and arising at 45 degree angle from stigmatic surface, length of upper parts exceeding that of lower parts. *Flower at first night of anthesis*, inner tepals white, outer staminodia tipped blue-violet; *second night anthesis*, inner tepal adaxial surface pink, inner tepals pale pink at base, white or pink towards the apex, outer staminodia dark pink for basal two-thirds of their length, white then violet towards the apex, inner staminodia pink at base. *Seeds* c. 300 per fruit, 12–13 × 16–17 mm, globose with a prominent raphe (especially when dry), dark brown to black, surrounded by a mucilaginous aril.

**Distribution and Conservation status** *Victoria boliviana* Magdalena and L. T. Sm. is restricted to Bolivia and the flood plains of the Llanos de Moxos, Mamoré watershed, identified as a Centre of Plant Diversity and Endemism (Site SA24) (Beck and Moraes, 1997). These area is considered by Langstroth Plotkin (2012). Moxos is surrounded by the forests of the Upper Madeira basin and is an area of largely open vegetation – herbaceous wetlands, grasslands, savannas, and woodlands (Beck, 1983, 1984; Langstroth Plotkin, 2012). Images (not included in the minimum calculation of the EOO or AOO) suggest that *V. boliviana*’s range extends further west (natural or cultivated) to Rurrenabaque. We estimated a minimum and maximum EOO and AOO. The maximum range was based on the potential habitat across the Llanos de Moxos region (including geographical information from public unverified images) and verified herbarium collections and iNaturalist images. The minimum range was based on the coordinates of herbarium collections and iNaturalist images alone. We estimated that the EOO of *V. boliviana* ranges between 8,006 km^2^(minimum) and 33,151 km^2^ (maximum), falling close to the thresholds of the Vulnerable category under criterion B. We estimated that the AOO of *V. boliviana* ranges from 32 km^2^ (minimum) to 2,000 km^2^ (maximum), falling between the Endangered and Vulnerable categories. There are less than five known locations for *V. boliviana*. We could find no information about population fragmentation but believe that *V. boliviana* may be vulnerable to fluctuations in flooding and drought throughout the year. For example, Beni Department has been recently affected by seasonal floods, fires and droughts due to El Niño and La Niña climate events (Vásquez, 2015). A recent increase in agriculture-lead deforestation has been documented along the Trinidad-Santa Cruz highway (Langstroth Plotkin, 2012), to the south of known *V. boliviana* populations and satellite images from Google Earth Pro and i-Terra suggest extensive deforestation along the edges of roads (i-Terra, 2021; NASA, 2021; GoogleEarth, 2022) which we use to infer an active decline in habitat quality.

Taking a precautionary approach and based on the small EOO and AOO, small number of locations (5), and continuing decline in habitat, we assess *V. boliviana* as Vulnerable (**VU**), according to criteria B1ab(iii)+B2ab(iii).

**Notes.** *Victoria boliviana* sp. nov. has the largest observed leaves of the three species, with laminae > 3 m in length having been observed. The abaxial surface of the upturned rim surface varies between individual plants in the same locality from dark maroon to very pale, almost “white” green, a characteristic not found in other species. Prickles on abaxial outer tepal surfaces are absent or very few, and if present are not confined to the lower portion of the outer tepals unlike *V. cruziana* where the smaller number of prickles are confined to the lower one-third of the abaxial tepal surface.

*Victoria boliviana* is the only species of Victoria whose carpellary appendages have upper portions that are longer than the lower portions. In addition, *V. boliviana*’s stigmatic chambers are the shallowest of the three *Victoria* species. Haenke saw *Victoria* plants in Yacuma in 1801, during the Malaspina expedition (Gickelhorn, 1966, p. 105; Ibañez Montoya, 1984), but no identifiable description is available and no voucher has been found. d’Orbigny (1840, p. 57) reported seeing this species on the banks of the Mamoré river in 1832 and mistakenly assigned this species to *V. amazonica* when publishing his description of *V. cruziana*.

Further investigations and surveys are required to better understand the species’ current range, population fluctuations and habitat and thus better predict the impacts of the threats identified.

Field observations of the flowers from a single population in the Llanos de Moxos suggest that whilst pollinated by beetles, *V. boliviana* sp. nov. flowers may host fewer individuals of pollinators than *V. amazonica*, only 4–10 individuals being observed in the flowers of the former, compared to > 20 in the flowers of the latter. This could be due to a lower density of pollinators in their area of occupation.

**Additional Material –** BOLIVIA. **Beni:** Santa Ana del Yacuma, –65.4236, –13.74, –/6–7/1845, *Bridges, T. s. n.* (K); Cercado: Laguna Suarez 5 km sur de la ciudad de Trinidad, –64.864167, – 14.872222, 08/05/2019, *Magdalena, C. Melgar, D. G., Salazar, C. D., Alvarez, C., Gutierrez, G., Arias, J. 154* (German Coimbra Sanz Herbarium, Jardin Botanico Municipal); Moxos, pasando el Río Mamoré, cerca al puente del Río Tijamuchi a lado del camino, –65.145278, –14.851111, 09/05/2019, *Magdalena, C. Melgar, D. G., Salazar, C. D., Alvarez, C., Gutierrez, G., Arias, J.155* (Herbario German Coimbra Sanz Jardín Botanico Municipal).

**Victoria cruziana** Orb., *Ann. Sci. Nat., Bot., sér. 2*, 13: 57 (January 1840). Type: Bolivia [Argentina], Corrientes, banks of the Paraná river, Arroyo de San José, beginning of 1827, d’Orbigny *s.n.* (lectotype: P (P02048598*) (designated by de Lima et al., 2021); isolectotypes: P (P02048599).

**FIGURE 11:**
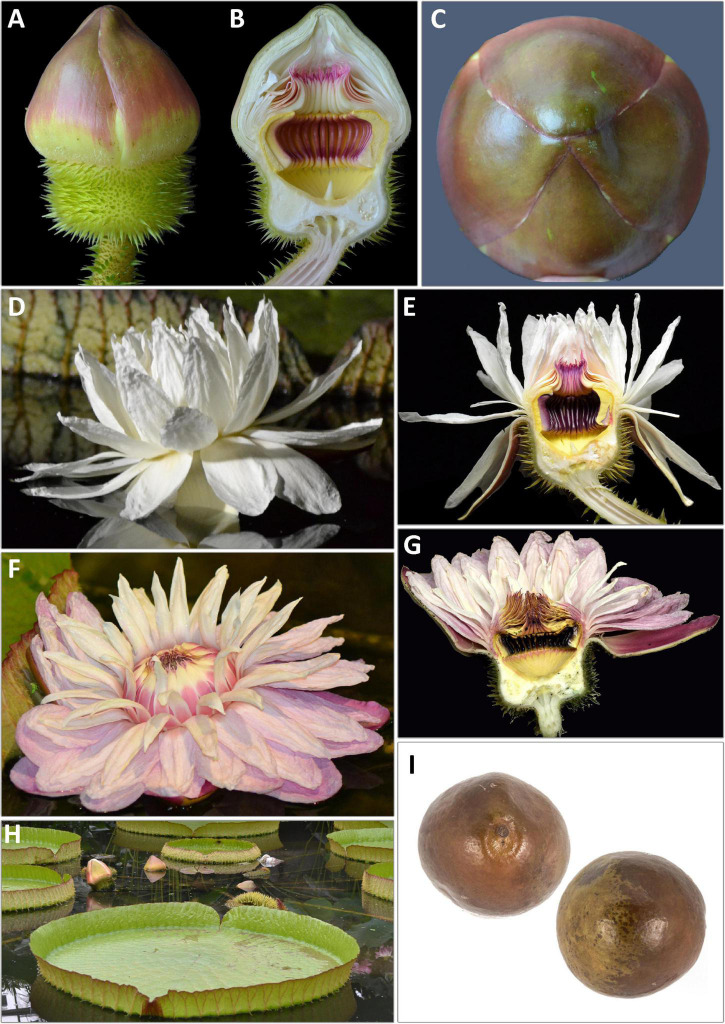
*Victoria cruziana* **(A)** bud whole, **(B)** bud L.S., **(C)** bud from above, **(D)** first night flower, **(E)** first night flower L.S., **(F)** second night flower, **(G)** second night flower LTS, **(H)** habit, and **(I)** seed. **(A–D,F,G)** (photo LTS), **(I)** (CM) cultivated RBG Kew, **(D,E)** cultivated Denver Botanic Gardens (LTS).

Vernacular names: Irupé (yrupé), yacare yrupé, naanók lapotó (poncho del otany), maíz de agua, Santa Cruz Waterlily, Victoria regia. Figures 1C, 3G–I, 6, 11.

*Victoria regia* var. *cruziana* (Orb.) G. Lawson, *Proc. & Trans. Roy. Soc. Canada* 6(4): 109 (1889)

*Euryale brasiliana* Steud., *Nomencl. Bot.* [Steudel], ed. 2. 1: 617 (November 1840).

*Euryale policantha* Rojas Acosta, *Cat. Hist. Nat. Corrientes* 65 (1897).

*Euryale bonplandia* Rojas Acosta, *Cat. Hist. Nat. Corrientes* 151 (1897).

*Victoria cruziana* f. *trickeri* Henkel ex Malme, *Acta Horti Berg.* 4(5): 12. 1907 (“Trickeri”), *nom. nud.*

*Leaves* up 2.4 m broad, adaxial surface of lamina green, abaxial surface of lamina green or dark blue-green, radial and reticulate ribs yellow or green; leaf margins form a high rim 8–10% of leaf length, rim ± perpendicular to or slightly recurved over adaxial surface at base, flared outwards at top (sigmoid in profile), abaxial surface of rim green or tinged maroon, hairs 1–3 mm, simple, multicellular, 10–15 segmented. *Flower* bud broadly ovoid, concave just before apex, up to 30 cm diameter at second-night anthesis. Ovary 7–10 cm diameter, outer surface covered in prickles 1–22 mm (dried), prickles abruptly tapering from c. half their length to sharp apex; hairs absent or present, where present 0.1–12 mm; inner surface of ovary with moderately concave stigmatic surface, rounded to triangular in longitudinal profile, ridged with lines corresponding with 25–38 radially arranged locules, each containing 20–25 ovules 1.5–1.8 mm (fresh). Outer tepals 4, 10–13 × 4–9 cm when fresh, abaxial surface green and/or tinged maroon abaxially prickles absent or present, where present up to 100 per tepal, prickles tapering abruptly at their midpoint to a sharp point, 1–10 mm (dried), distributed up to lower one third of surface, hairs absent or present, where present 0.1–1 mm. Inner tepals 7–10 × 1.5–9 cm (fresh), innermost all white both in bud and during first-night anthesis, crinkled in appearance, turning pale to dark pink on second-night; outer staminodia, 6–7 × 1–1.5 cm, thick, rigid, apiculate; stamens 4–6 × 0.5–1 cm inner staminodia > 50, 4–6 × 0.5 cm; base of lower parts of carpellary appendage flat, arising from stigmatic surface at 45 degree angle, cuneate, length of upper parts not exceeding that of lower parts. *Flower at first night of anthesis:* all inner tepals white, outer staminodia tipped pink; at *second night anthesis*, outer tepal adaxial surface pink, inner tepals pale or dark pink at base, white or pink towards apex, outer staminodia dark pink for basal two-thirds of their length, white then pink towards apex, inner staminodia pink at base. Seeds, c. up to 1000 per fruit, 7–9 × 8–10 mm, globose, raphe faintly visible, brown to black, surrounded by a mucilaginous aril.

### Distribution and Conservation Status

*Victoria cruziana* f. *mattogrossensis taxon incertum* has hitherto been included within *Victoria cruziana* until this study. Because our genomic analyses are limited with respect to the rank of this taxon and of its relationship to the other species, we have not considered forma *mattogrossensis taxon incertum* as conspecific with *V. cruziana* for the purposes of an extinction risk assessment.

*Victoria cruziana* is restricted to the Paraná river basin and tributaries, from Paraguay to Argentina, and possibly Bolivia. Based on the maximum potential habitat of wetlands (including Esteros de Ibera National Park Wetlands) and a combination of verified herbarium collections and iNaturalist images we estimate the EOO of *V. cruziana* to be between 46,563 and 132,945 km^2^. This exceeds the threshold for a threatened category under criterion B (IUCN Standards and Petitions Committee, 2019). We calculate the AOO to be 120 km^2^, although an upper estimate based on the extent of the river may exceed 2,000 km^2^ (but not more than 3,000 km^2^). This would assess *V. cruziana* as between Endangered and Vulnerable categories. There are more than 10 locations but no information about population fragmentation. We infer a continuing decline in habitat quality due to the increasing frequency of droughts, the abstraction of water, deforestation (Caivano and Calatrava, 2021; Comisión Nacional de Actividades Espaciales, 2021) and big hydroelectric dams. For example, Itaipu is one of the largest dams in the world (Stevaux et al., 2009) and lies within the *V. cruziana* range. Whilst the AOO upper estimate is close to the threshold for VU, there are more than sufficient criteria for a threatened category under criterion B, and we therefore assess *V. cruziana* as LC.

We recommend further documentation of the distribution and size of *V cruziana* populations and investigations into their fluctuation through time as we believe that it may be vulnerable to an increase in the frequency and severity of droughts associated with climate change and increased sedimentation caused by the construction of large dams within its habitat (Stevaux et al., 2009).

**Notes.** *Victoria cruziana* forms the proportionately highest leaf rims of all the species, and these are always slightly recurved over the flat part of the lamina, flaring out at the top. The concavity of the outer tepals before their apex gives the bud a pinched-in appearance. Prickles are absent or occur on the outer tepal abaxial surface, but only up to one-third of their length from the base. In this species, hairs which are sometimes present on the lower outer tepal abaxial surface and ovary are the only ones large enough to see without magnification. At first night anthesis, inner tepals have a crinkled appearance.

*Victoria cruziana* f. *mattogrossensis taxon incertum* was described by Malme based on material present in the spirit collection of the Swedish Museum of Natural History comprising two jars of the same gathering. S07-84 comprises a longitudinal section through the ovary and perianth showing clearly the distribution of prickles and their form on both the ovary and outer tepals, also the morphology of the carpellary appendages; S07-85 comprises sections of the petiole apex and either the petiole or pedicel.

Both are preserved in excellent condition. S07-84 was selected as lectotype as it displayed a greater number of diagnostic morphological features.

*Victoria cruziana* f. *mattogrossensis taxon incertum* was described from, and material corresponding to it has only been observed from the Pantanal, in Bolivia, Brazil and Paraguay in the Uruguay river basin.

Phylogenomic data were unable to confirm whether the material sampled represents a distinct evolutionary lineage (see section “Discussion”). It may be that further sampling and research supports the recognition of this name as a distinct taxon.

**Material Examined.** ARGENTINA. **Chaco:** 1st De Mayo, Laguna en Chacra, al lado del arroyo Ine, –58.849444, –27.416389, 02/03/2006, *Mulgura de Romero, M. E., Anisko, T., Harbage, J., Illarrage, H.4249* (SI); San Fernando, Entre Barranqueras e Isla Antequera, –58.87944, –27.416389, 18/03/1967, *Krapovickas, A. and Cristobal, C. L. 12752* (MO). **Corrientes:** Capital, Riachuelo, off Ruta 12 ca 17 km S of Corrientes, –58.749444, –27.554444, 06/04/1982, *Schinini, A. Wiersema, J. H. 2243* (MO); Esquina, Isla Correntina frente a curuzu – Chali, en el Paraná medio, –59.630833, –30.341944, 10/04/1968, *Burkart, A., Troncoso, N. S., Guaglianone, E. R. and Palacios, R. A. 26963* (SI); San Roque, R. Santa Lucia, –58.738056, –28.576944, 27/02/1957, *Pedersen, T. M. 4486* (K, MO); Bella Vista, Cruce Ruta Nacional 12 Ey Puente Sobre Lel Río Santa Lucia, –58.72, –28.57, 12/04/2008, *Mulgura de Romero, M. E., Aniśko, T., Belgrana, M. J. and Harbage, J. 4474* (SI); Dep. Esquina, Río Guayguiraro, –59.56, –30.374444, 26/02/1974, *Quarin, C., Schinini, A. Gonzales, J. M., Ishikawa, A. 2196* (K). **Santa Fe:** La Capital, Ruta Nacional 168 y Puente no. 9, Sobre Arroyo mini, al E-SE de la Ruta Prov. 1. W, –60.575833, –31.672778, 12/04/2008, *Mulgura de Romero, M. E., Anisko, T., Belgrana, M. J. and Harbage, J. 4477* (SI). BOLIVIA. **Santa Cruz:** Angel Sandoval, channel at southern end of Laguna Mandiore, –57.483333, –18.216667, 16/07/1998, *Ritter, N., Crow, G. E., Garvizu, M. and Crow, C. 4562* (LPB, MO) [f. mattogrossensis], *Ritter, N., Crow, G. E., Garvizu, M. and Crow. C.* 4560 (LPB, MO). BRAZIL. **Mato Grosso du Sul:** Corumba, Cacimba da Saude, – 57.664167, –18.99833, 13/12/2002, *Avellar, A. L. F. 13* (COR) [f. Mattogrossensis]; Ladario, terminal da Branave, no porto de Ladario, –57.584722, –19.0225, 12/08/1994, *Sanches, A. L., Bortolotto, J. M., Damascenos Jr., G. 44* (COR) [f. mattogrossensis]; Corumba, Ladario, CODRASA Brejo (swamp), – 57.516389, –19.021111, 27/11/2004, *Souza Jr., A. F., and Siqueira, C. S. 39* (COR) [f. Mattogrossensis]; Corumba, Ladario, –57.580556, –19.001111, –/07/1894, *Anon s.n. [G. O. A. Malme] S07-785, S07-784*

(S). PARAGUAY. **Distrito Capital:** L’Assumption [in the swamps], –57.604167, –25.256667, 17/03/1875, *Balansa, R. 523* (K, P). **Central:** Piquete Cue, –57.666667, –25.166667, 29/11/2000, *Zardini, E. M. and Guerrero, L. 55181* (MO); Bay of Ascuncion, wetlands, –57.61922, –25.273333, 15/03/1988, *Ericsson, K. 577* (MO). **Neembucu:** Yataity, –58.040556, –26.788611, –/03/1975, *Walter, M. A. 86* (K); Pilar Garden Club S, –58.276389, –26.867778, 28/01/2005, *De Egea Juvinel, J., Pena-Chocarro, M.., Vera, M., Torres, M.. and Elsam, R. 738* (MO). **Presidente Hayes:** Riacho Pucu., –57.083333, –24.666667, 20/08/2000, *Zardini, E. M and Guerrero, L. 54765* (MO); 12/02/1987, *Sparre 2363/51* (P).

### Taxon Incertum

*Victoria cruziana* f. *mattogrossensis* Malme, *Acta Horti Berg.* 4(5): 12. 1907. Type: [Brazil, Matto Grosso do Sul, near Corumba] *Anon s.n.*[G.O.A. Malme] 1894 [July] [S (spirit collection) (lectotype (selected here): S (S07-784*); isolectotype: S (S07-785*)]

### Excluded Names

*Victoria argentina* Burmeister, *Reise durch die La Plata Staaten 2: 5* (1861).

*Victoria fitzroyana* Hort. Ex Loudon, Encyc. Pl. Suppl. Ii. 1388. = *Nymphaea gigantea* Hook.

## Data Availability Statement

The datasets presented in this study can be found in online repositories. The names of the repository/repositories and accession number(s) can be found below: NCBI GenBank – SUB11368210.

## Author Contributions

CM, DGM-G, CDS, and GG-S conducted exploratory fieldwork and set up local partnerships in Bolivia which led to the conception of this study. LTS, CM, AKM, OAP-E, NASP, and AA conceived and designed the research, with contributions from OM and SD. CM, DGM-G, CDS, and GG-S conducted fieldwork. AKM conducted taxonomic and nomenclatural research. LTS created the botanical illustrations. LTS, CM, and AKM collected and analysed the morphological data. CM and LTS collected geographical data. NASP conducted wet lab work for DNA sequencing. OAP-E and NASP conducted *in-silico* molecular analyses. RN conducted conservation status assessments. IJL and SM conducted genome size wet lab work and analyses. SM conducted chromosome counts. CM cultivated living material that was used for horticultural observations, illustrations, the chromosome count and the estimation of genome size. SB, DGM-G, GR-G, and CDS contributed type material and plant tissue. AA contributed analytical tools and reagents. LTS and OAP-E produced the figures, with contributions from NASP. AKM, LTS, and NASP wrote the manuscript with contributions from CM, RN, OAP-E, and IJL. All co-authors reviewed and approved the submitted manuscript.

## Conflict of Interest

The authors declare that the research was conducted in the absence of any commercial or financial relationships that could be construed as a potential conflict of interest.

## Publisher’s Note

All claims expressed in this article are solely those of the authors and do not necessarily represent those of their affiliated organizations, or those of the publisher, the editors and the reviewers. Any product that may be evaluated in this article, or claim that may be made by its manufacturer, is not guaranteed or endorsed by the publisher.

## Footnotes

1
http://victoria-adventure.org/

2
https://chlorobox.mpimp-golm.mpg.de/geseq.html
