# Supplementary material for: Revised Species Delimitation in the Giant Water Lily Genus Victoria (Nymphaeaceae) Confirms a New Species and Has Implications for Its Conservation
Source: Front Plant Sci. 2022 Jul 4;13:883151. doi: 10.3389/fpls.2022.883151 (PMC9289450; doi:10.3389/fpls.2022.883151)
Supplement: Supplementary file 3 [file Data_Sheet_1.PDF]

Running Title: Species delimitation in the Giant Water Lily genus *Victoria*

SUPPLEMENTARY FIGURES AND TABLES

Figure S1 Principal component analysis of *Victoria* nuclear genomic dataset comprising 16 individuals based on 436,329 genotype likelihoods obtained after skimming for a minor allele frequency (*maf*) threshold of 0.1.

Figure S2. Flow histogram showing peaks of *Victoria boliviana* used to estimate genome size. Flow histogram was produced by FlowMax software. 1 = G_1 peak of the internal standard *Petroselinum Crispum* with a known genome size of 1C= 2.22pg. 2 = G_1 peak of the sample, *Victoria boliviana* sp. nov. 3 = G_2 peak of *Victoria boliviana* sp. nov.

Figure S3 Metaphase chromosomes of *Victoria boliviana* stained with aceto-orcein showing a chromosome count of 2n = 24.

Table S1 Details of samples used for molecular work: taxonomy, geographical origin and collection details.

Table S2 Geographical observations (biological collections, iNaturalist).

Table S3 Amazon, Tocantins and Paraná water basins from which *Victoria* has not been documented.

Table S4 Morphological observations from biological collections.

Table S5 Morphological observations from iNaturalist images.

Table S6 Summary of polymorphic sites differentiating intraspecific *Victoria* genomes

Table S7 Samples used for molecular work. Tissue source and totals of raw reads before and after trimming.

Table S8 Samples used for molecular work. Summary statistics for reads mapped to *V. cruziana* plastid.

Table S9 Samples used for molecular work. Summary statistics for reads mapped to 37,470 *V. cruziana* transcript sequences.

Table S1. Details of samples used for molecular work: taxonomy, geographical origin and collection details.

Sample ID	Taxon	Country	Locality	Collector	Collector No.	Herbarium
NPNY4	*Nymphaea ampla*	-	-	Magdalena		K
NPNY6	*V. amazonica*	Guyana	Akuri Pond	BGA Gardens 150 Guyana Expedition	G70	Adelaide
NPNY7	*V. amazonica*	Brazil	Para, Municipio de Oriximiná lago Uraría, SW of Oriximiná, across Rio Trombetas	Davidson C. & Martinelli G.	10241	MO
NPNY8	*V. amazonica*	Peru	Loreto, Padre Isla in Río Amazonas below Iquitos	Gentry A.H. et al.	22133	MO
NPNY11	*V. amazonica*	Brazil	Amazonas, Ipixuna, Rio Cróa	Quinet A. et al.	1582	K
NPNY12	*V. amazonica*	Bolivia	Santa Cruz, Angel Sandoval	Ritter N.	4562	MO
NPNY13	*V. amazonica*	Brazil	Amazonas, Rio Solimões, S bank near Carreiro	Steward W.C. & Ramos J.F.	P20211	K
NPNY14	*V. amazonica*	Guyana	North Rupununi Karanamlon Ranch, Crane Pond, off of Rupunini River	Te, T.S.; Vonow, H.P.; Sawtell, C.B.	137	Adelaide
NPNY15	*V. amazonica*	Brazil	Amazonas, 100 km lower reaches from Manaus	Tsugaru S.	43774	MO
NPNY18	*V. cruziana*	Paraguay	Neembucu, Pilar Garden Club	Egea J. et al.	738	MO
NPNY20	*V. cruziana*	Argentina	Corrientes, Capital	Schinini A. & Wiersema J.H.	2243	MO
NPNY21	*V. cruziana*	Paraguay	Presidente Hayes, frente Trinidad	Sparre & Vervoorst	2363	P
NPNY22	*V. cruziana*	Paraguay	Presidente Hayes	Zardini E.M. & Guerrero L.	54765	MO
NPNY23	*V. cruziana*	Paraguay	Central	Zardini E.M. & Guerrero L.	55181	MO
NPNY24	*V. boliviana* sp. nov.	Bolivia	12 km fro fatima To Ignacio and TrinitadBeni, road to San Ignacio	Magdalena et al.	1	USZ
NPNY25	*V. boliviana* sp. nov.	Bolivia	Laguna Suarez (Near Trinitad)	Magdalena et al.	2	USZ
NPNY26	*V. boliviana* sp. nov.	Bolivia	Trinidad Cuartel Trinitdad Inicio PuebloBeni, Trinidad, road 9	Magdalena et al.	3	USZ
NPNY27	*Victoria* sp.	Brazil	Matto Grosso, Rio de Barbado	Weddell H.A.	3422	P

Table S2. Geographical observations of wild populations of *Victoria*. Observations took the form of herbarium collection records (collector name in italics) or identifiable iNaturalist records (denoted by *).

**Name**	**Longitude**	**Latitude**	**Observation identifier**	
amazonica	-55.9025	-1.812778	*Davidson Martinelli* 10241	
amazonica	-55.910278	-1.803889	*Martinelli* 6945	
amazonica	-64.813056	-7.229772	*Prance* 8016	
amazonica	-59.141944	-3.216111	*Tsugaru et al* B769	
amazonica	-73.163611	-3.651944	*Gentry & Jarmillo* 22133	
amazonica	-61.8525	-1.158333	*Martinelli* 17700	
amazonica	-60.728056	-3.260278	*Tsugaru* B1069	
amazonica	-55.961111	-4.229167	*Brogim* 4	
amazonica	-66.126667	-10.903611	*Ritter* 4170	
amazonica	-55.083333	-1.833333	*Jangoux & Riberio* 1647	
amazonica	-72.556667	-7.745278	*Quinet et al* 1582	
amazonica	-70.0625	-4.103611	*Gentry &Daly* 18351	
amazonica	-76.166667	-3.75	*Vasquez* 3684	
amazonica	-61.503889	-1.570833	*Adalardo-Oliveira* 2645	
amazonica	-54.043333	-2.011944	*Lima* 503	
amazonica	-54.70684	-2.45007	*Spruce* s.n.	
amazonica	-58.27775556	4.394033333	*Schomburgk s.n.*	
amazonica	-59.30778	3.745278	*Maas* 7727	
amazonica	-59.806667	-3.168889	*Steward & Ramos* P20211	
amazonica	-59.123847	3.93153	45279665	*
amazonica	-59.295456	3.748394	15852553	*
amazonica	-59.295326	3.748124	635983	*
amazonica	-59.308258	3.750822	9451814	*
amazonica	-59.329548	3.69647	525202	*
amazonica	-59.347986	3.674892	32511297	*
amazonica	-59.343042	3.660387	61074769	*
amazonica	-59.790001	3.371667	9174107	*
amazonica	-54.623053	-2.474785	26382442	*
amazonica	-54.728823	-2.39235	8683881	*
amazonica	-54.733572	-2.385142	19502338	*
amazonica	-54.750062	-2.347232	26329395	*

amazonica	-54.850917	-2.266025	24958095	*
amazonica	-54.854393	-2.266102	6710083	*
amazonica	-54.862289	-2.300331	12194823	*
amazonica	-54.858586	-2.298044	25938393	*
amazonica	-54.858428	-2.298087	25915077	*
amazonica	-54.85732	-2.299208	7632580	*
amazonica	-59.741197	-3.134012	18828530	*
amazonica	-59.755167	-3.096333	40632911	*
amazonica	-59.845318	-3.05866	11952160	*
amazonica	-59.850001	-3.067468	44770752	*
amazonica	-59.849381	-3.070873	44544043	*
amazonica	-59.897204	-3.070358	4249757	*
amazonica	-59.932127	-3.001659	50185207	*
amazonica	-59.939652	-3.00291	11884045	*
amazonica	-59.941063	-3.006066	16512534	*
amazonica	-59.940208	-3.007255	27909284	*
amazonica	-59.940394	-3.007239	49738977	*
amazonica	-59.940418	-3.007371	36870328	*
amazonica	-59.956536	-3.009903	11909720	*
amazonica	-60.021731	-3.119027	51618250	*
amazonica	-60.038513	-3.101099	56829000	*
amazonica	-59.997447	-3.192778	29787728	*
amazonica	-60.003149	-3.215419	22201090	*
amazonica	-60.025526	-3.207815	22144955	*
amazonica	-59.99776	-3.2233	13250517	*
amazonica	-60.003891	-3.2275	46690161	*
amazonica	-60.050729	-3.268442	19004119	*
amazonica	-60.091765	-3.252047	37350813	*
amazonica	-60.123172	-3.294003	34061547	*
amazonica	-60.185644	-3.301105	10494779	*
amazonica	-60.187102	-3.287892	20537321	*
amazonica	-59.858514	-3.266262	29478806	*
amazonica	-59.833482	-3.461615	25707022	*
amazonica	-60.011936	-3.541866	13781155	*
amazonica	-61.850057	-1.772505	35238613	*

amazonica	-60.697753	-2.739008	47054543	*
amazonica	-60.56488	-2.901896	38273109	*
amazonica	-58.76674	-5.979301	1116550	*
amazonica	-58.505233	4.247119	15745912	*
amazonica	-59.123847	3.93153	45279665	*
amazonica	-64.645058	-2.723533	17205305	*
amazonica	-64.848926	-3.063124	42986245	*
amazonica	-64.84193	-3.079977	30941538	*
amazonica	-67.11904	-4.488923	12617884	*
amazonica	-69.900513	-4.2259	6373562	*
amazonica	-69.927998	-4.289593	20802253	*
amazonica	-69.935907	-4.203165	21262099	*
amazonica	-69.942595	-4.197528	26111456	*
amazonica	-69.943078	-4.21292	37212476	*
amazonica	-69.975528	-4.179212	10546880	*
amazonica	-69.975528	-4.179212	10546881	*
amazonica	-69.993355	-4.165763	19442883	*
amazonica	-70.017257	-4.1801	33111156	*
amazonica	-70.073629	-4.16216	26017905	*
amazonica	-70.060944	-4.1465	19505602, 20802253	*
amazonica	-70.063887	-4.142199	51747676	*
amazonica	-70.056853	-4.110197	22241172	*
amazonica	-70.208708	-4.31636	13654259	*
amazonica	-70.205832	-3.865026	39279556	*
amazonica	-70.255695	-3.81027	23067255	*
amazonica	-70.297685	-3.810987	18327780	*
amazonica	-70.355642	-3.788867	58907430	*
amazonica	-70.346869	-3.812669	20853375	*
amazonica	-72.515478	-3.473687	10236407	*
amazonica	-72.765488	-3.348924	4101345	*
amazonica	-72.844849	-3.444883	821386	*
amazonica	-72.869884	-3.461312	16366927	*
amazonica	-72.861328	-3.529869	835181	*
amazonica	-73.040403	-3.551092	14688494	*
amazonica	-73.130705	-3.624705	57510649	*

amazonica	-73.016999	-3.491679	3683800	*
amazonica	-73.062	-3.51	18857631	*
amazonica	-73.117709	-3.564369	7193201	*
amazonica	-73.132088	-3.606303	50636039	*
amazonica	-73.316049	-3.752315	31466499	*
amazonica	-73.335022	-4.119948	44866058	*
amazonica	-73.199947	-4.186663	32659562	*
amazonica	-73.39193	-4.264329	45373341	*
amazonica	-73.348568	-4.263766	35679795	*
amazonica	-73.318778	-4.303537	42857333	*
amazonica	-73.28265	-4.298399	9675454	*
amazonica	-73.26442	-4.326718	128022	*
amazonica	-73.419998	-4.513739	41477934	*
amazonica	-73.429417	-4.529185	44128190	*
amazonica	-73.454127	-4.529635	54312531	*
amazonica	-73.452788	-4.512862	62183827	*
amazonica	-73.539508	-4.49316	19421939	*
amazonica	-73.626277	-4.556509	7354070	*
amazonica	-73.644312	-4.609328	24415081	*
amazonica	-73.555847	-4.667159	43258612	*
amazonica	-73.564539	-4.692089	7736153	*
amazonica	-73.598313	-4.690977	7736136	*
amazonica	-74.38	-5.05	19541409	*
amazonica	-75.442657	-5.2063	39408977	*
amazonica	-74.172133	-2.294842	5074735	*
amazonica	-72.818525	-7.600695	61086794	*
amazonica	-72.690425	-7.647397	35378749	*
amazonica	-72.578998	-7.736687	21152434	*
amazonica	-72.568611	-7.760556	33785950	*
amazonica	-72.562992	-7.764506	33411887	*
amazonica	-72.555623	-7.755204	59978746	*
amazonica	-72.552134	-7.750067	33824102	*
amazonica	-72.550535	-7.745338	33513251	*
amazonica	-72.553563	-7.743627	34224087	*
amazonica	-59.295251	3.747129	67206405	*

amazonica	-58.767702	3.860997	70239327	*
amazonica	-59.35512	3.663073	70363817	*
boliviana	-65.0452	-12.821	62457426	*
boliviana	65.2082	-14.874078	18398989	*
boliviana	-64.904	-14.8161	67432069	*
boliviana	-66.6166667	-14.2	*Beck* 15173	
boliviana	-65.423611	-13.74	*Bridges* s.n.	
boliviana	-64.8642	-14.8722	*Magdalena and Melgar* 154	
boliviana	65.1453	-14.8511	*Magdalena and Melgar* 155	
cruziana	-58.87944	-27.416389	*Krapovickas & Cristobal* 12752	
cruziana	-59.630833	-30.341944	*Burkart & Troncoso* 26963	
cruziana	-57.60416667	-25.256667	*Balansa* 523	
cruziana	-58.040556	-26.788611	*Walter* 86	
cruziana	-59.56	-30.374444	*Quarin Schinini* 2196	
cruziana	-58.738056	-28.576944	*Pedersen* 4486	
cruziana	-58.749444	-27.554444	*Schinini* et al 2243	
cruziana	-57.61922	-25.273333	*Ericsson* 577	
cruziana	-57.083333	-24.666667	*Zardini & Guerrero* 54765	
cruziana	-57.666667	-25.166667	*Zardini & Guerrero* 55181	
cruziana	-58.72	-28.57	*Mulgura* 4474	
cruziana	-58.849444	-27.416389	*Mulgura* 4249	
cruziana	-58.276389	-26.867778	*De Egea Juvinel* 738	
cruziana	-57.489972	-25.085978	47990535	*
cruziana	-57.47865	-25.109547	25703910	*
cruziana	-57.475683	-25.162297	47596112	*
cruziana	-58.152755	-26.172912	41597516	*
cruziana	-58.145833	-26.1675	35772480	*
cruziana	-58.166289	-26.223596	38465815	*
cruziana	-58.199722	-26.24	12253146	*
cruziana	-58.422198	-26.960643	16249075	*
cruziana	-58.731746	-27.094628	19516477	*
cruziana	-58.832971	-27.408756	56708356	*
cruziana	-58.972225	-27.452991	19756472	*
cruziana	-59.588299	-30.021989	88305	*
cruziana	-59.930634	-30.597365	47310054	*

cruziana	-59.930634	-30.597365	47310055	*
cruziana	-60.092748	-31.0944	20905920	*
cruziana	-60.486073	-31.51626	21270698	*
cruziana	-60.652987	-32.129617	19686973	*
cruziana	-60.62929	-32.886575	44398386	*
cruziana	-60.61669	-32.921326	43510141	*
sp 'mattogrossensis'	-5.664167	-18.99833	*Avellar* 13	
sp 'mattogrossensis'	-57.584722	-19.0225	*Sanches Bortolotto* 44	
sp 'mattogrossensis'	-57.516389	-19.021111	*Souza* 39	
sp 'mattogrossensis'	-59.979875	-15.805618	*Weddell* 3422	
sp 'mattogrossensis'	-57.483333	-18.216667	*Ritter* 4562	
sp 'mattogrossensis'	-57.803606	-16.339997	8504852	*
sp 'mattogrossensis'	-57.803575	-16.340022	8504850	*
sp 'mattogrossensis'	-56.709709	-17.287934	47077077	*
sp 'mattogrossensis'	-56.809759	-17.373684	20327463	*
sp 'mattogrossensis'	-56.775965	-17.363482	40360119	*
sp 'mattogrossensis'	-56.776222	-17.36345	8054907	*
sp 'mattogrossensis'	-56.776173	-17.363467	8788584	*
sp 'mattogrossensis'	-56.776153	-17.36348	31305311	*
sp 'mattogrossensis'	-56.77617	-17.363575	31279820	*
sp 'mattogrossensis'	-56.776115	-17.36371	31660321	*
sp 'mattogrossensis'	-56.775931	-17.364182	31250944	*
sp 'mattogrossensis'	-56.775856	-17.36452	31232741	*
sp 'mattogrossensis'	-56.773131	-17.364933	22693198	*
sp 'mattogrossensis'	-57.552688	-18.748286	35167856	*
sp 'mattogrossensis'	-56.776397	-17.3623	40363312	*
sp 'mattogrossensis'	-56.775967	-17.363878	40360114	*
sp 'mattogrossensis'	-56.775883	-17.363572	40360115	*
sp 'mattogrossensis'	-56.77594	-17.363778	40360116	*
sp 'mattogrossensis'	-56.77608	-17.363468	40360126	*
sp 'mattogrossensis'	-56.776017	-17.363553	40360124	*
sp 'mattogrossensis'	-56.776232	-17.63335	8217338	*
sp 'mattogrossensis'	-56.7762	-17.363298	8475313	*
sp 'mattogrossensis'	-56.77617	-17.363788	7559115	*
sp 'mattogrossensis'	-56.77617	-17.363668	30940933	*

sp 'mattogrossensis'	-56.776458	-17.36205	8705900	*
sp 'mattogrossensis'	-57.432597	-17.655786	25627078	*
sp 'mattogrossensis'	-57.432597	-17.655786	25627081	*

Table S3. Amazon, Tocantins and Paraná water basins from which *Victoria* has not been documented.

**River basin**	**River system**	**Country**
Amazon	Magdalena	Colombia
Amazon	Cauca	Colombia
Amazon	Arauca	Colombia
Amazon	Meta	Colombia
Amazon	Voupes	Colombia
Amazon	Apopuris	Colombia
Amazon	Caqueta	Colombia
Orinoco	Guaviare	Colombia
Amazon	Putumayo	Colombia
Amazon	Uyacali	Peru
Amazon	Peruvian portion of the Rio Blanco (tributary Rio Negro)	Peru
Amazon	Peruvian portion of the Madre de Dios	Peru
Amazon	Vaupes	Brazil
Amazon	Rio Negro	Brazil
Amazon	Rio Branco (Acre)	Brazil
Amazon	Rio Branco flood plain	Brazil
Amazon	Putumayo	Brazil
Amazon	Japura	Brazil
Amazon	Trombetas	Brazil
Amazon	Paru	Brazil
Amazon	Jari	Brazil
Amazon	Madeira	Brazil
Amazon	Teles	Brazil
Amazon	Piros	Brazil
Amazon	Arinos	Brazil
Amazon	Xingu	Brazil
Amazon	Iriri	Brazil
Tocantins	Araguaia	Brazil
Tocantins	Tocantins	Brazil
Paraná	upper course of the Paraguay river (Pantanal to Asuncion)	Argentina, Paraguay

Supplementary Table S4. Morphological observations from biological collections.

[Table S4 morphological observations.xls to be linked here]

Supplementary Table S5. Morphological observations from iNaturalist images.

[Table S5 morphological observations.xls to be linked here]

Table S6. Summary of polymorphic sites differentiating *Victoria* plastid genomes. LCB denotes 'local collinear block' – assigned by the mauveAligner algorithm.

LCB number	Sites	Sites with gaps	Total monomorphic sites	Total polymorphic sites	Singleton mutations	Parsimony informative sites	Alleles private to *V. boliviana*
LCB1	51,458	734	50,675	49	45	4	2
LCB2	39,156	376	38,733	47	38	9	2
LCB3	20,125	19,533	19,381	25	21	4	4
LCB4	25,281	379	24,902	61	61	0	0
full plastid	136,020	21,022	133,691	182	165	17	8

Table S7. Samples used for molecular work. Tissue source and raw sequence read counts before and after read trimming.

Sample ID	Leaf tissue source	Total raw reads	Total trimmed paired reads	Total trimmed collapsed reads
NPNY4	fresh	7700068	648524	6883146
NPNY6	fresh	7554596	1341579	6874838
NPNY7	herbarium	9078078	65088	6300086
NPNY8	herbarium	6324963	56240	4139503
NPNY11	herbarium	11150233	69607	7098393
NPNY12	herbarium	6277459	60129	4363992
NPNY13	herbarium	7432859	46874	4815434
NPNY14	fresh	12562419	1378200	11967990
NPNY15	herbarium	7836548	80579	3845751
NPNY18	herbarium	4764159	35074	2818630
NPNY20	herbarium	3912151	35362	1984198
NPNY21	herbarium	9714532	113974	5486855
NPNY22	herbarium	5880535	57026	3189822
NPNY23	herbarium	6912504	73269	4278868
NPNY24	fresh	14036804	518350	12282597
NPNY25	fresh	11207799	1303473	10895428
NPNY26	fresh	11421150	1900554	10584322
NPNY27	herbarium	5601236	53134	3355957

Table S8. Samples used for molecular work. Summary statistics for reads mapped to *V. cruziana* plastid genome.

Sample	Plastid: mapped paired	Plastid: mapped collapsed	Plastid: mapped, merged, no PCR duplicates	Plastid: % PCR duplicates	Plastid completeness (% bases genotyped)	Plastid: Average depth of coverage
NPNY4	1289805	7662341	1419689	84.1	99.99	1476.8
NPNY6	1215488	2668339	1392281	64.2	100	1490.2
NPNY7	4784	938112	171383	81.8	99.92	89.9
NPNY8	3556	383260	160896	58.4	99.95	77
NPNY11	6195	2623542	236083	91	99.96	119.9
NPNY12	16753	4106353	274044	93.4	100	189.7
NPNY13	11529	4962329	301185	93.9	99.98	177.9
NPNY14	907083	2973585	1120012	71.1	100	1233.7
NPNY15	37841	7149852	309282	95.7	99.98	129.1
NPNY18	4733	1150409	170904	85.2	99.7	74.1
NPNY20	4941	1482116	194333	86.9	99.9	81.3
NPNY21	48894	9154036	304703	96.7	100	121.1
NPNY22	8667	1536190	178641	88.4	99.32	71.1
NPNY23	19820	5428498	296956	94.5	100	155.2
NPNY24	207023	3848507	512118	87.4	100	612.5
NPNY25	421028	1465893	701700	62.8	100	801.1
NPNY26	46812	105602	128051	16	99.99	138.2
NPNY27	10875	2704038	236504	91.3	99.99	113.3

Table S9. Samples used for molecular work. Summary statistics for reads mapped to 37,470 *V. cruziana* transcript sequences, where endogenous content is an estimate based on this set of nuclear data only and weighted according to the relative proportions of collapsed and paired reads.

Sample	Transcript set: mapped paired	Transcript set: mapped collapsed	Transcript set: merged, no PCR duplicates	Transcript set: % PCR duplicates	Transcript set: Endogenous content	Transcript set: Average depth of coverage
NPNY4	3599208	17174212	6569649	91.6	45.7	34.5
NPNY6	22262587	46993018	17684028	82.6	57.3	32.9
NPNY7	59258	24546205	1695154	78.5	11.9	3.5
NPNY8	8634	1437644	423997	68.4	1.1	3.9
NPNY11	59504	27914612	2838367	89.9	11.4	4.8
NPNY12	53779	18733255	2270864	87.9	14	4.5
NPNY13	42360	20464093	2760393	86.5	12.6	4.5
NPNY14	23269460	83069201	17437373	91.7	61.7	30.6
NPNY15	123106	31682742	2649880	86.7	16.5	3.6
NPNY18	50516	18446924	2456101	78.7	0.3	3.9
NPNY20	33349	12581037	1843034	92	14.6	3.2
NPNY21	130326	41249211	3309246	89.9	19.4	4.3
NPNY22	67218	22069096	2242239	88	16.8	4.2
NPNY23	66363	23099373	2769817	86.9	15	4.2
NPNY24	7116441	86743932	7838319	93.1	45.7	17.0
NPNY25	19287259	65732261	14787122	70.7	57.3	26.5
NPNY26	31487809	71271281	22144242	96.3	68.6	32.8
NPNY27	52179	19412570	2558916	83.6	11.9	3.5
