# Supplementary figures and images for: Revised Species Delimitation in the Giant Water Lily Genus Victoria (Nymphaeaceae) Confirms a New Species and Has Implications for Its Conservation
Source: Front Plant Sci. 2022 Jul 4;13:883151. doi: 10.3389/fpls.2022.883151 (PMC9289450; doi:10.3389/fpls.2022.883151)

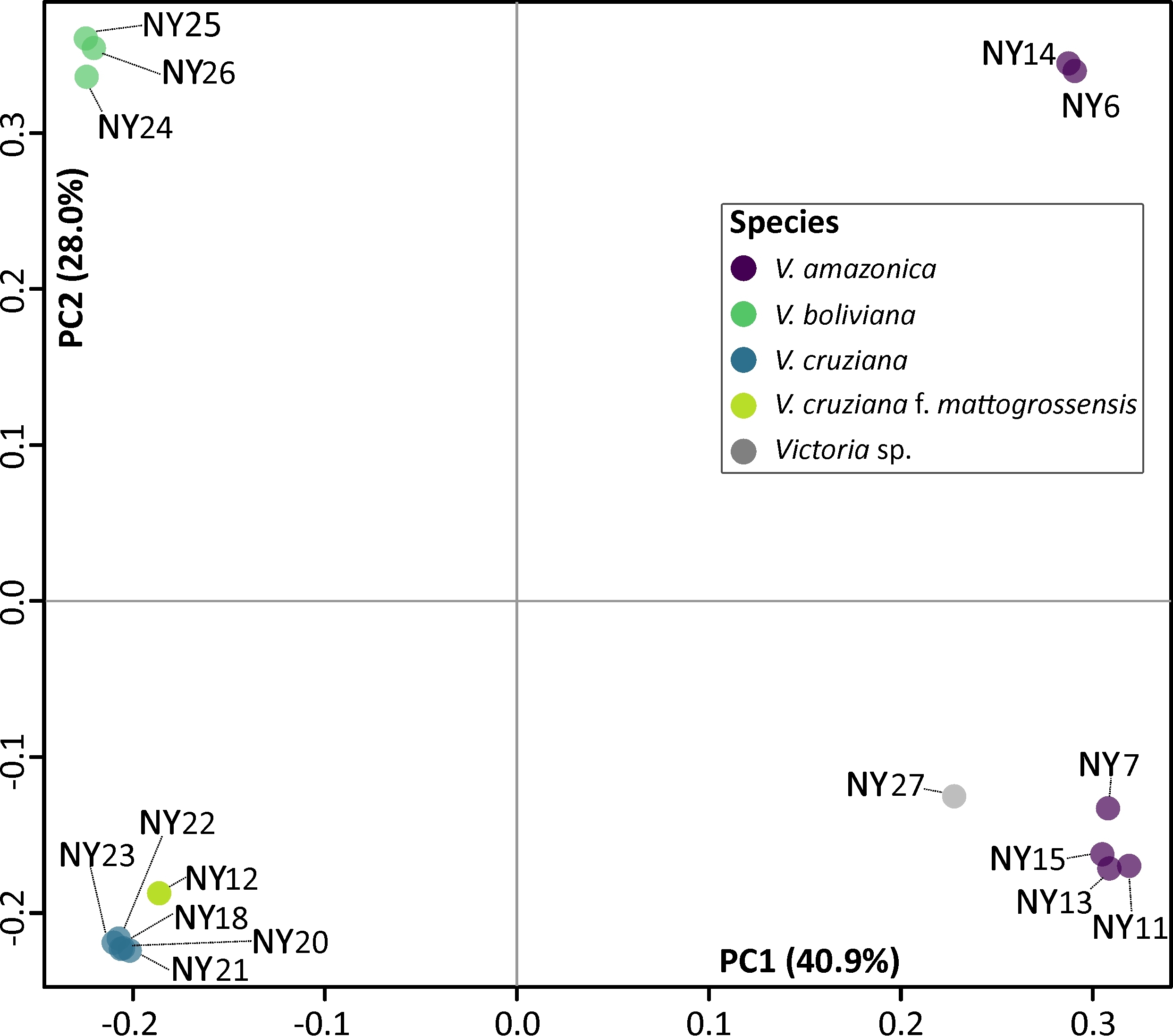

Supplement: Supplementary file 4 [file Image_1.JPEG]

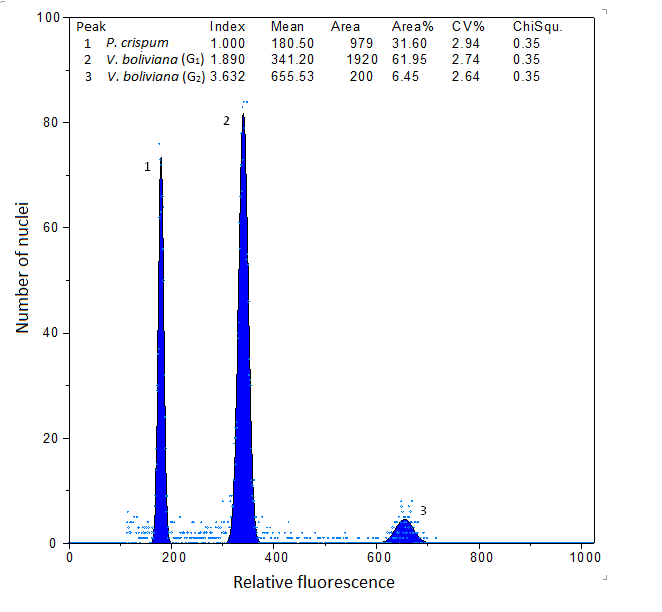

Supplement: Supplementary file 5 [file Image_2.PNG]

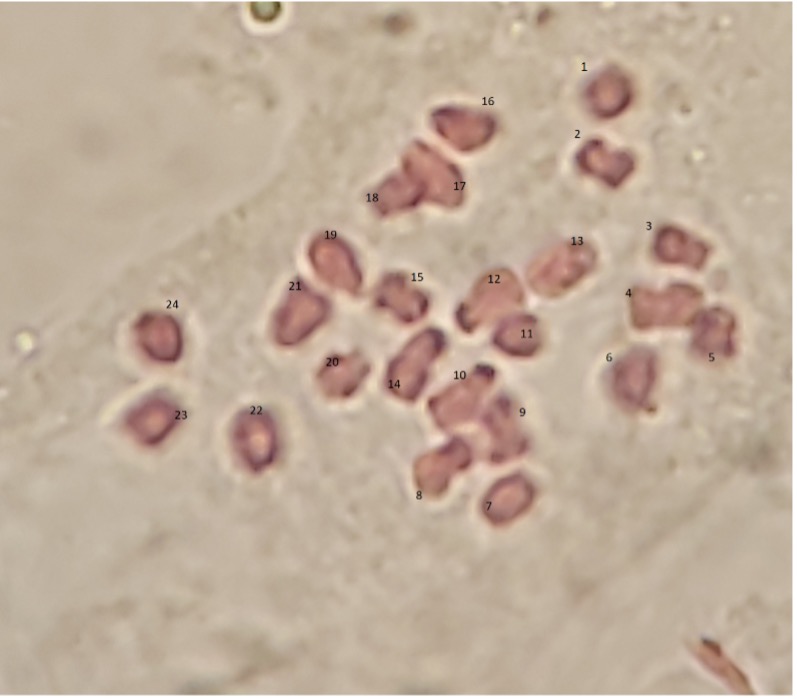

Supplement: Supplementary file 6 [file Image_3.JPEG]
